# Cortical dynamics of perception as trains of coherent gamma oscillations, with the pulvinar as central coordinator

**DOI:** 10.1186/s40708-024-00235-w

**Published:** 2024-08-20

**Authors:** J. Farineau, R. Lestienne

**Affiliations:** 1 System Analyst, Cholet, France; 2 Honorary Research Director at CNRS, Paris, France

**Keywords:** Cortical dynamics, Pulvinar, Synchrony, Gamma cycle, Cortical coordination, Inhibition mechanisms

## Abstract

Synchronization of spikes carried by the visual streams is strategic for the proper binding of cortical assemblies, hence for the perception of visual objects as coherent units. Perception of a complex visual scene involves multiple trains of gamma oscillations, coexisting at each stage in visual and associative cortex. Here, we analyze how this synchrony is managed, so that the perception of each visual object can emerge despite this complex interweaving of cortical activations. After a brief review of structural and temporal facts, we analyze the interactions which make the oscillations coherent for the visual elements related to the same object. We continue with the propagation of these gamma oscillations within the sensory chain. The dominant role of the pulvinar and associated reticular thalamic nucleus as cortical coordinator is the common thread running through this step-by-step description. Synchronization mechanisms are analyzed in the context of visual perception, although the present considerations are not limited to this sense. A simple experiment is described, with the aim of assessing the validity of the elements developed here. A first set of results is provided, together with a proposed method to go further in this investigation.

## Introduction

Driving in a big city at the busiest times of the day is a real challenge for the brain. Many visual objects are discriminated and tracked, only a small proportion of them consciously, all to ensure a safe trajectory and anticipation of any potential danger: obstacles, erratic changes in the trajectory of another vehicle, etc.

From the very first level, V1, primary visual cortex, a clear dissociation between visual components is established, based at this stage on color, orientation, luminance, spatial frequency, and motion [10, 64]. This segregation is repeated at subsequent levels, giving rise to a diversity of processing paths where, at each level, components recombine or further divide. So how can the simultaneous perception of hundreds of visual objects operate when, for each of them, the pieces of information are disseminated in distinct areas of the cortex, sharing the same substructures? The mechanisms involved are multiple and complex. We will limit ourselves to identifying the circuits producing synchronous bursts of activation for the components of the same visual object, and analysing the interaction modalities associated with these circuits. We will focus on the purely temporal aspects. The principles of sensory analysis and segmentation are not considered here.

To analyze the timing of the sensory chain, we briefly review the facts reported on the structural and temporal levels regarding the cortical and thalamo-cortical interactions. Then, based on these elements, we propose a model for these interactions, production of gamma bursts, synchronization of related cortical columns and propagation to downstream cortical areas.

The thalamus is known to play a key role in the production of gamma oscillations, acting like a cortical pacemaker [25, 41, 45, 55]. We will focus here on the pulvinar, the thalamic structure associated to the higher-order visual areas. We will investigate the synchronous activity widespread over distant cortical areas, the role of the pulvinar is here fundamental.

Due to the crumpled structure of the cortex, the length of fibers linking the thalamus to the cortex is highly variable. Despite this variability, it is observed a very low and nearly constant latency for these links, thanks to adapted myelination [44]. This aspect is fundamental in the capacity of the pulvinar to play the role of central coordinator, in a tight temporal relationship with the associated cortical columns.

This central role of the pulvinar is ensured in mutual association with cortical layers 4 to 6 [4, 9, 41, 45]. Layer 5 is unique in this respect, as it is the source of subcortical projections. Beyond this major role, we will analyze here the equally important contribution of this layer to the synchronization process.

If we consider the exchange of information within the cortex, the supragranular layers appear to be the real carriers of these exchanges, given the enormous volume of physical links they represent. The volume of signal exchanges mediated by the pulvinar is almost negligible by comparison. The pulvinar cannot be considered a node of information exchange. In contrast, we shall see that the pulvinar perfectly plays the role of a central synchronization node, in close association with layers 4 to 6.

Based on the proposed model of interaction, we will analyze the global behavior, through the overall response time (RT). An interesting fact concerns the delay between the onset of a simple visual stimulus and the global activation of the frontal areas [7, 8, 48]. Even with simple cases, this delay is high, ~ 300 ms, but relatively deterministic, a surprising effect when one considers the variability of the latency introduced by each cell and the complexity of the sensory chain. There may be some link between RT and gamma frequency, whose rate is also deterministic in situation of observation. This proposition is analyzed here, completed by an experiment whose first results are consistent with the analysis outcomes. We will see that the statistics on RT make explicit the stability of the mean value.

Visual perception involves feedforward streams, processing, and top-down interactions [10, 13, 28, 54, 62, 69]. The way the information is processed at each stage to give rise to the activation of neurons assemblies will not be addressed here. We will mainly focus on the sequencing of the feedforward exchanges, in order to clarify their role in the timing structure of neuronal discharges. We will also partially address the top-down interactions as they have some influence on the timing of these feedforward streams even with such simple visual stimulus.

The term cortical stage (CS) will be introduced here to designate the set of cortical areas whose first bursts of activity are synchronous, following the occurrence of a visual stimulus. A wave of activation starts in V1, and gradually propagates to subsequent areas in the cortical hierarchy. Areas belonging to a same cortical stage are those whose activity starts in synchrony.

## Terminology

CS Cortical Stage

CT Cortico-Thalamic

DM DorsoMedial thalamic nucleus

IPSP Inhibitory PostSynaptic Potential

LGN Lateral Geniculate Nucleus

M1 Primary motor cortex

MGN Medial Geniculate Nucleus

MT Middle Temporal area

PFC Prefrontal Cortex

PM PreMotor cortex

RT Response Time

RTN Reticular Thalamic Nucleus

SD Standard Deviation

VA Ventral Anterior thalamic nucleus

VL Ventral Lateral thalamic nucleus

VPM Ventral Posterior Medial thalamic nucleus

## A technical analysis

Trying to understand the synchrony mechanisms underlying perception is a technical matter, based on the identification of each type of neuron involved, their temporal characteristics, and how they are coupled.

The propagation of visual signals is gradual, in the form of a relatively diffuse wave propagating within the visual cortex. This progression lasts ~ 300 ms before the massive activation of frontal areas in the case of conscious perception [7, 8, 32, 48]. Although relatively long, this delay is rather deterministic. One might have expected a wide range of fluctuations, with an equally wide range of mean values, given that the latency introduced by each neuron is itself highly variable. This is not the case. One of the main objectives here is to identify what justifies this travel time, starting with the analysis of the elementary module, referred to here as cortical stage (CS).

We will review the components of the cortical stage and analyze their particularities, dynamic behavior, as well as the interactions between the higher-order cortical areas and the associated thalamic structure, i.e. the pulvinar. This analysis will be conducted via a review of literature, gathering the facts reported about these topics, and after that we will build a model which fits with the related facts.

The sensory chain is made up of a succession of cortical stages (V1, V2, V4, and so forth for the visual perception). We will see how they are coupled, here mainly for the feedforward flow, and determine the links between the delay introduced by each cortical stage and the overall RT.

The last sections describe a simple experiment intended at characterizing the variability of the overall RT, and the first elements that we can draw from these parallel approaches.

## Structural facts

The following considerations focus on those elements that serve the present concern, in this case everything related to thalamo-cortical interactions, and the propagation of excitation and inhibition immediately following the onset of a stimulus. We need to keep in mind each structural particularity that has an incidence on the dynamic behavior. Most of these aspects are commonly known, but there are several details that are not so frequently evocated, although they do influence the physical interactions.

Each cortical column is composed of minicolumns mutually coupled through excitatory and inhibitory horizontal projections. Orthogonal to the forward cortical propagation path, lateral connections between cortical areas terminate in patches, 200 to 500 μm wide, separated by gaps of equal width [35].

The following facts were investigated by Thomson and Bannister [58], Thomson and Lamy [59], Thomson [60]; see also Markram et al., [33], Harris et al. [22].

**Fig. 1:**
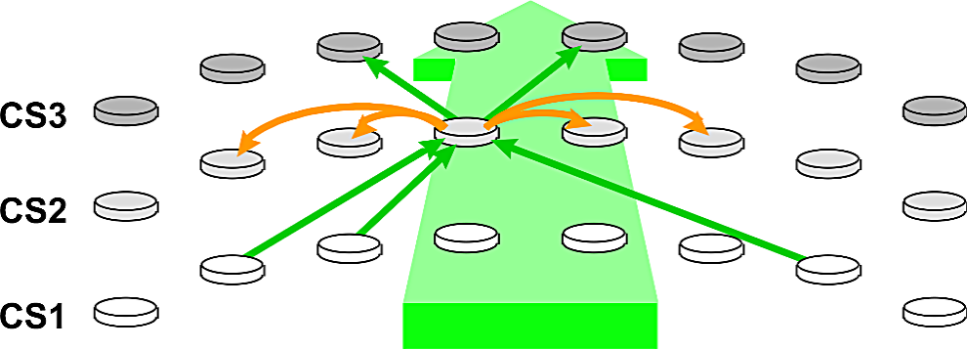
Symbolic representation of feedforward and lateral projections. CS1-3: simplified illustration of 3 successive cortical stages. Main green arrow: direction of feedforward propagation. Circular symbols: symbolized cortical patches, spaced as described by Mountcastle [35]. Green links: feedforward excitatory projections from one cortical stage to the next. Orange links: lateral projections (excitatory and inhibitory), with a strategic role in the synchronization process as we shall see

For projections interconnecting pyramidal cells, layer 3 axons target layer 5 via dense, focused projections, and extend laterally as horizontal projections. Long projections terminate in localized patches within the supragranular layers. The same organization applies to layer 5, with major implications as we shall see. Figure 1 symbolizes these lateral projections.

While afferents to the pulvinar are collaterals of layer 5 and 6, layer 5 collaterals do not innervate the RTN (Reticular Thalamic Nucleus). RTN inhibitory interneurons innervate relay cells via their soma and proximal dendrites. These couplings are fast and precise in time. In contrast, the feedforward cortical projections are received by the relay cells through smaller, more distal inputs.

Regarding the long horizontal projections from layer 6, the unmyelinated excitatory projections are doubled by inhibitory projections, which are myelinated. Consequently, the horizontal propagation of inhibition is generally faster than the spread of excitation.

Thalamo-cortical projections connect to layer 4 stellate cells. Each of these excitatory interneurons projects to 300–400 pyramidal neurons in layer 3, and symmetrically an equivalent number of these interneurons converge on each cell in layer 3. In addition, each stellate cell innervates and is innervated by 200 of its peers. We will here refer to this organization as cluster of stellate cells, where excitatory signals are summed and diffused, within the cortical minicolumn and the neighboring ones.

Each layer 6 projection innervates a relatively narrow zone of the RTN and pulvinar, corresponding to the associated thalamocortical projection but also overflowing it. The layer 6 projection is therefore not strictly limited to the relay cells which are connected to layer 4 of the same column; it also influences the adjacent relay cells [26]. To be also noted: inhibitory interneurons of layers 5 and 6 project back to the upper layers.

Concerning the thalamic relay cells, while the first-order cells (of the LGN) relay subcortical afferents, the higher-order cells relay cortical afferents, from layer 5 [5]. Still regarding the structural facts, we must recall the important point mentioned in the introduction: despite the highly variable length of fibers linking the thalamus to the cortex, the latency is very low and nearly constant for these links (typically 2 ms), thanks to adapted myelination [44].

All these elements regarding layer 4, layer 6, and the links between the pulvinar and the associated cortical columns, indicate that this organization is not bound to exchanges of information. As developed by Saalman et al. (2012) [42], they are essential for the promotion of neuronal synchrony between cortical areas.

**Fig. 2:**
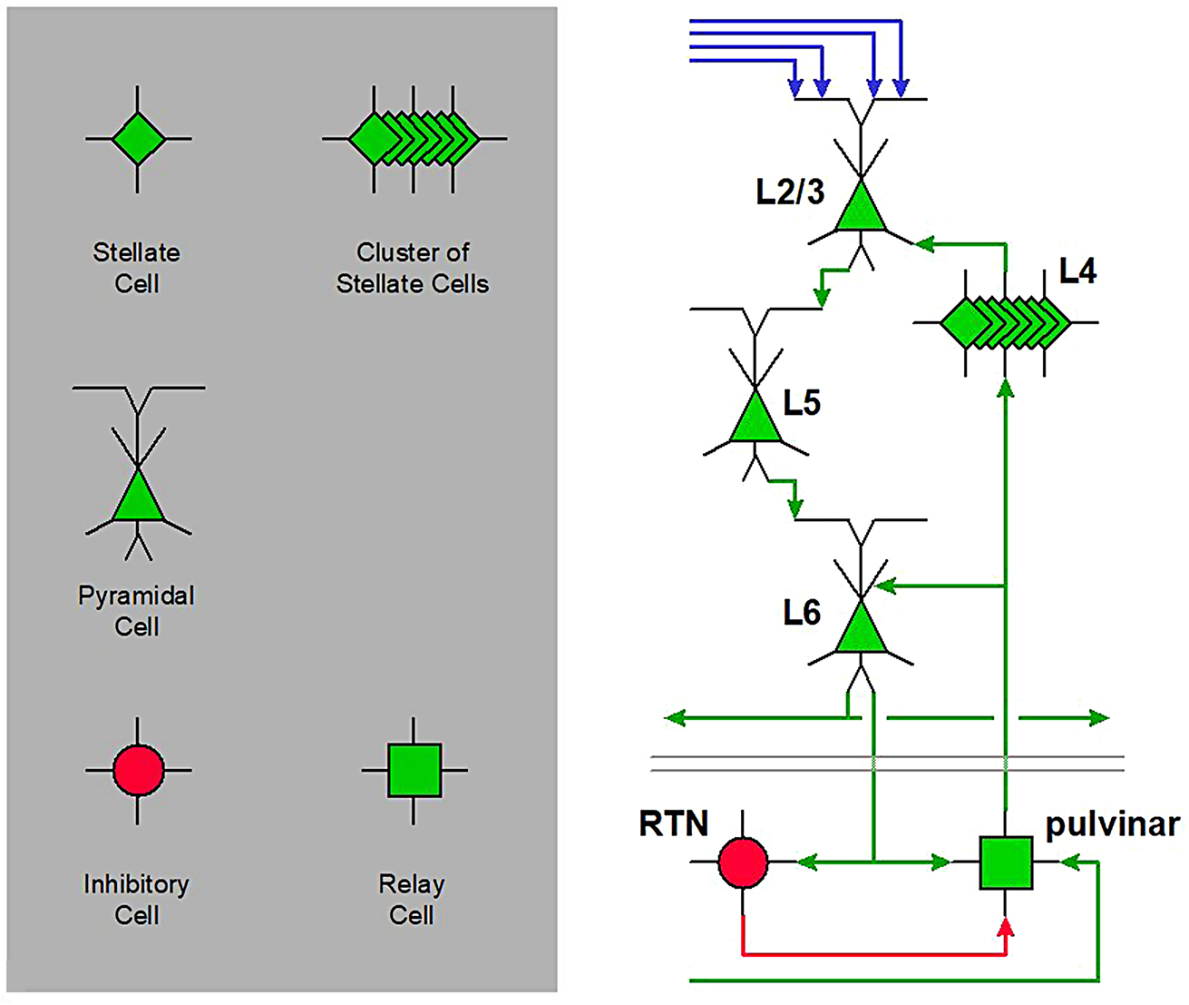
Basic diagram of main higher-order thalamo-cortical interactions and list of associated symbols. Green links: excitatory, red links: inhibitory, blue links: cortico-cortical projections, excitatory. This diagram presents the elements of the cortical column, their relationships and interactions with thalamic structures, aspects which will be progressively detailed

Based on these elements, Fig. 2 illustrates the physical interactions within the cortical layers and with the pulvinar. related to synchrony. We will follow on with the timing of each interaction.

**Fig. 3:**
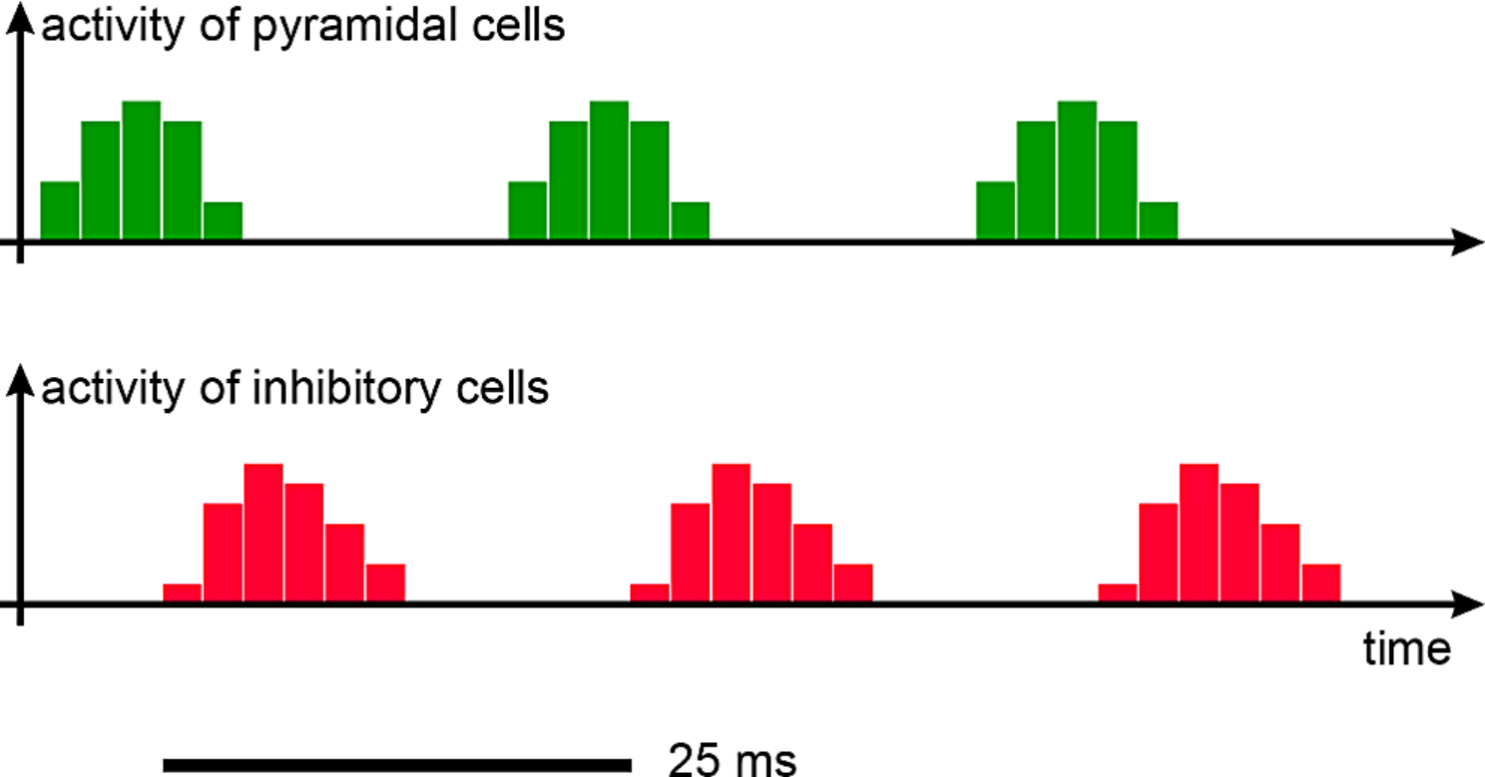
Sketch of activity of pyramidal cells versus activity of the cortical inhibitory interneurons (inspired from Fries et al., [16]. Each excitation phase is immediately followed by an inhibition phase, due to inhibitory interneurons innervated by the pyramidal cells, resulting in a limited time window for excitation

**Fig. 4:**
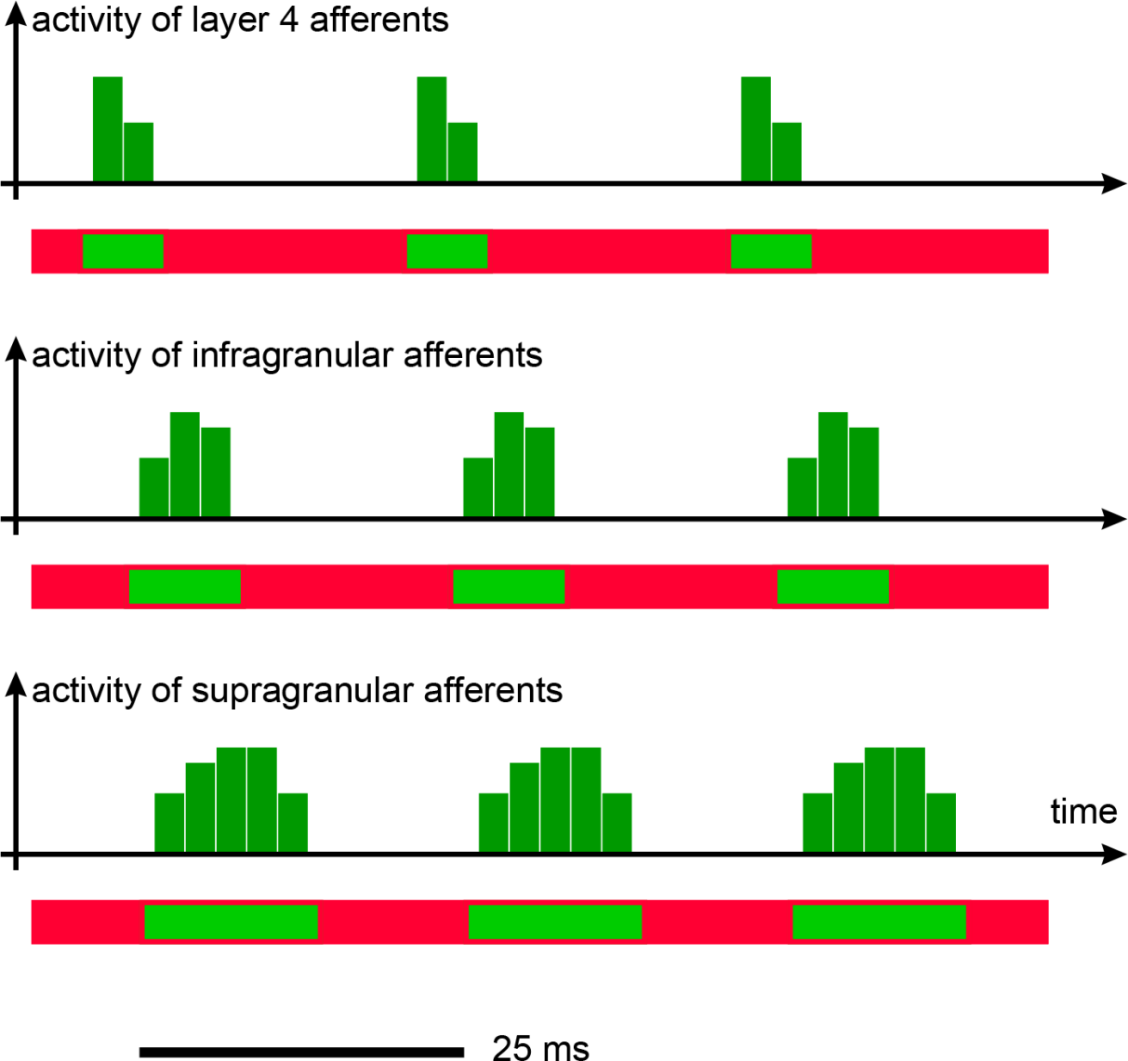
Sketch of activity of synchronous afferents and corresponding time windows (horizontal bars), based on Wilent & Contreras, [71]. The first signals, the most precise in time, are those relayed by layer 4. The activity of the other layers is slightly delayed and spread out over time. Time windows are consistent with this temporal staggering, as inhibition precisely follows activation within each layer (horizontal bar, red: inhibition is active; green: no inhibition)

### Temporal facts

Timing is the key factor, whatever the angle from which cortical mechanisms are considered: the exchange and propagation of spikes, the coding of information, the precise phasing between excitation and inhibition, and the wave of activation that follows the occurrence of a stimulus [7, 12, 16, 18, 26, 30, 31, 39, 41, 49–51, 55, 57, 66, 70–72].

Within the cortical column, inhibitory interneurons are innervated by pyramidal cells. They fire ~ 3 to 4 milliseconds after the associated pyramidal cells (Fig. 3, inspired from Fries et al., [16] see also Wehr and Zador, [70].

Pyramidal cells respond to excitatory inputs received within a specific time window, until inhibitory actions override excitations, allowing the most salient features to be extracted from a stimulus [4, 16, 47, 61]. The weakest afferents are ignored as their spikes, slightly delayed, exceed this time window.

Higher-order cortical exchanges follow two paths: the direct path, and the path mediated by the pulvinar [4, 36, 42, 43, 45]. These streams are organized as cyclical waves, with a gamma rhythm.

The first active cells are those in layer 4. Due to the clustering effect described for the stellate cells, the afferent spikes present in the time window are summed. Inhibition starts within 3 ms, limiting the time window for integration of excitatory inputs [17]. Cortico-cortical signals synchronous with the spikes received by layer 4 are fed forward, and signals that are not in phase (moreover at a different frequency) are ignored [16, 17]. This scheme provides an important answer to the initial question of interweaving of signals in the cortex for each visual object. Any non-coherent signal, potentially related to another object, is ignored.

Returning to the analysis of time course within the cortical column [71]:

- Activation starts at layer 4.
- Follows the activation of pyramidal cells in layers 3, then 5, and subsequently 2 and 6.
- While the time window is narrow at layer 4 for the integration of excitatory inputs, the time window is wider for the supra- and infra-granular layers, enabling these layers to integrate inputs over a larger time window.

The timing is coherent between any cortical stage and the next one: if related to a same visual element, the incoming spikes share a similar time structure as in Fig. 4. They are all active in phase.

## The synchronization scheme

Based on these facts, a major question concerns now the way in which distant areas belonging to a same cortical stage are synchronized, and thus can operate in mutual synchrony for a same visual object.

This synchronization may result from the interactions just described, simply based on the signals received from the preceding cortical stage. However, with such scheme, the longest cortico-cortical projections from the preceding cortical stage would result in excitation phases that would be delayed with respect to the shorter projections, to the detriment of synchrony and with a cumulative effect, from stage to stage.

We hypothesize that the lateral projections of layer 5 could be the source of synchrony between distant areas. We have mentioned that inhibitory projections are faster than excitatory ones. Let us consider a set of cells which get active for a given stimulus, within a same cortical stage. The activation delay is not strictly identical for all cells. Thus, the fastest cells provoke first the inhibition of the slower ones, through the inhibitory projections. For a same stimulus, this effect is benefic as it avoids a time spreading for the spikes fed to the subsequent cortical stage.

Immediately after this phase of inhibition, the excitatory projections produce the depolarization of these slowest cells. Thus, these cells are facilitated, and they can trig precisely at the next cycle, this time in phase with the leading cells. The role played by the pulvinar for this effect of synchronization will be described just after.

This modality is compatible with long projection distances. This would result in a precise replication of the gamma cycle even for distant cortical columns, justifying the role of central coordinator mentioned for the pulvinar.

These elements illustrate the complementarity between the two ways of synchronization [26]:

- Local synchronization mediated by layer 6, with layer 6 CT projections extending locally beyond the thalamic source, and reciprocally through the layer 4 clusters of stellate cells described by Thomson and Lamy [59], diffusing locally.
- Synchronization by the horizontal lateral projections from layer 5 to distant patches, i.e. fast inhibitory links and slower excitatory links, described by Thomson and Bannister [58].

## The pacemaker role of the thalamus

At Fig. 3, we can see a gap between the end of inhibition and the next activation: inhibition ceases at the cortical level, but the next activation does not immediately follow.

The pulvinar plays the role of cortical pacemaker [41, 45]. According to this principle, the observed time gap may not be due to the inhibition produced within the cortical column itself, but to the inhibition effective at the pulvinar level. This is the point on which we will now focus.

We have described the projections from layer 6 to the RTN, and the role of thalamo-cortical interactions in cortical synchronization. The effect of collaterals innervating the RTN could thus be at the origin of the time gap observed between the end of inhibition and the next activation. The advantage of such an organization is the centralized synchronization of the cortical columns involved in the same sensory event. The central position of the pulvinar and the low latency thalamo-cortical links support this, as does the mutual coupling between neighboring RTN inhibitory interneurons, based on fast direct gap junctions.

**Fig. 5:**
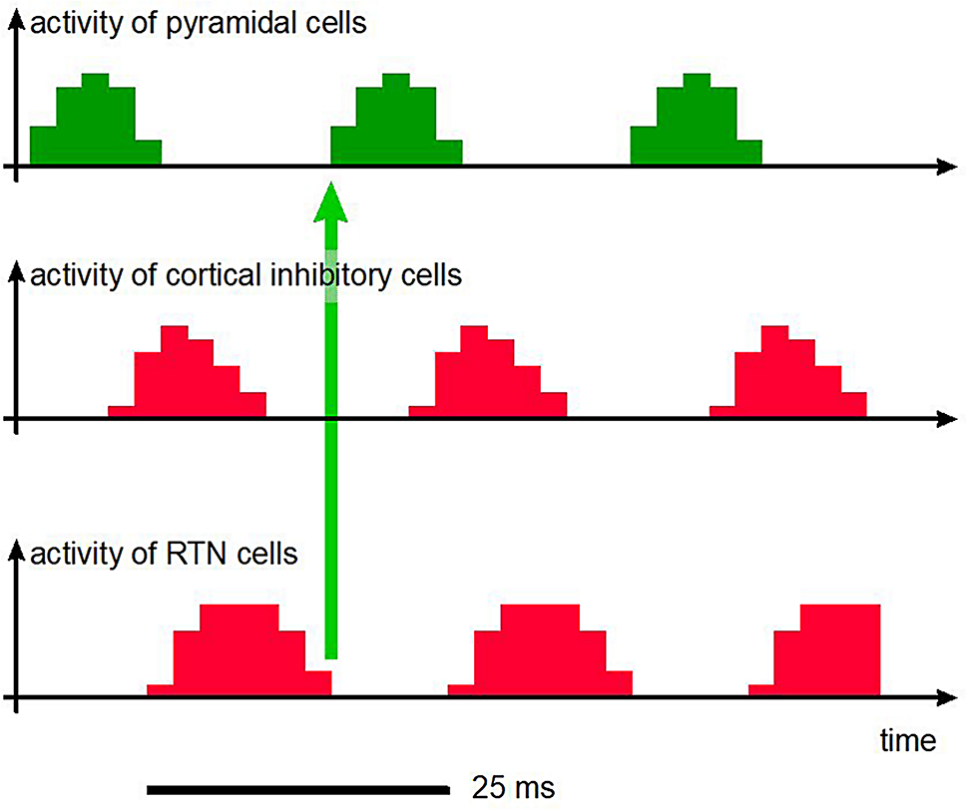
Illustration of the pacemaker role of RTN cells in the generation of gamma cycles. Activity is first suspended by the inhibitory cells within the cortical column, then by the RTN. The next cycle can occur only at the end of the inhibition phase imposed by the RTN (green arrow). Adjacent cortical columns are associated with adjacent RTN cells, mutually coupled by gap junctions, enabling synchronous operation of the whole, at the same rhythm of gamma cycle

RTN cell clusters may therefore act as pacemakers, through their action on the associated relay cells for the synchronization of signals that are fed to cortical columns: activity within the cortical column ceases under the action of the cortical inhibitory cells, and the next cycle cannot be initiated as long as the RTN inhibitory cells are active, resulting in the production of synchronous gamma cycles (Fig. 5): as soon as inhibition ceases, it is like a set of gates that open in phase and give rise to synchronous activation of the cortical columns.

On this basis, is the gamma period identical for all the objects composing the visual scene? The typical cycle lasts 25 ms, the gamma frequency being typically 40 Hz in humans, for an attended visual task (e.g. [11, 55, 56]). A visual scene is generally made up of many visual objects. We can focus our attention on one object; the associated gamma period is ~ 25 ms for this main visual object. However, the period associated with other objects in the same visual scene differs significantly.

A simple test consists in observing a small square, flashing 20 ms every 60 ms. When we focus our gaze on this central object, we do not miss any occurrence of the stimulus, but if we shift our gaze away from the target while keeping our full attention on it, perception loses its systematic character. The cause could be a slower burst cycle for the peripheral vision. It is likely that each perceived visual object has its own rhythm, not deterministic due to mutual inhibitions between neighboring assemblies.

This digression on parallel asynchronous assemblies will be left aside. From now on, we will focus on a simple case, the perception of a single attended visual object, thus linked to the ~ 25 ms gamma cycle.

### The cortical stages

To summarize the above considerations, there are two parallel paths linking two successive cortical stages: a time accurate path based on layers 4, 5 and 6 in association with the pulvinar, for synchronization purposes, and a direct path linking layers 2 and 3 to the same layers of the next stage, carrying the massive signals that detail the specifics of the stimulus. We have also mentioned the primordial role of the gamma cycle, which enables distributed computations to operate on the same reference time frame, in synchrony.

As previously defined, the term cortical stage (CS) is used here to designate the cortical areas whose activation starts within the same gamma cycle, on occurrence of a simple, contrasted visual stimulus. Even in the case of simple visual tasks, the timing of cortical activation is flexible [23]. The notion of cortical stage depends on the nature of visual stimulus. In order to pursue the detailed analysis of sensory timing, we will consider the example of a simple stimulus, a bright square appearing at a fixed location, without transition, over a permanent dark background. This is also the type of stimulus on which we will base the experiment described later on. Activation within a cortical area can be very rapid for certain stimuli, but in the general case, activation is progressive, depending on lateral recruitment and top-down interactions. However, even for areas where activation is progressive, statistical analysis based on repeated sessions can give access to the precise instant of onset of activation, for any cortical area significantly involved in the perception. Without such fast and contrasted stimulus, we could not mention the cortical stage as defined here, as the activation would be too much progressive, with blurred overlap of starting phases. We estimate that with this visual stimulus, EEG recordings and statistical analysis, it should be possible to discern the cortical stages, i.e. the nodes of cortical activity, and to identify their timing, the sequence of activation of these nodes. We shall come back on this future phase.

For the time being, can we get at least an idea about the number of cortical stages composing the visual sensory chain?

Thirty-two areas have been identified for the visual system hierarchy, organized into 10 successive levels [63]. It is not certain that each of these 10 levels can be considered a cortical stage as defined here. The actual number of cortical stages is essentially linked to the general topology of recruitment via the lateral interactions described here before.

**Fig. 6:**
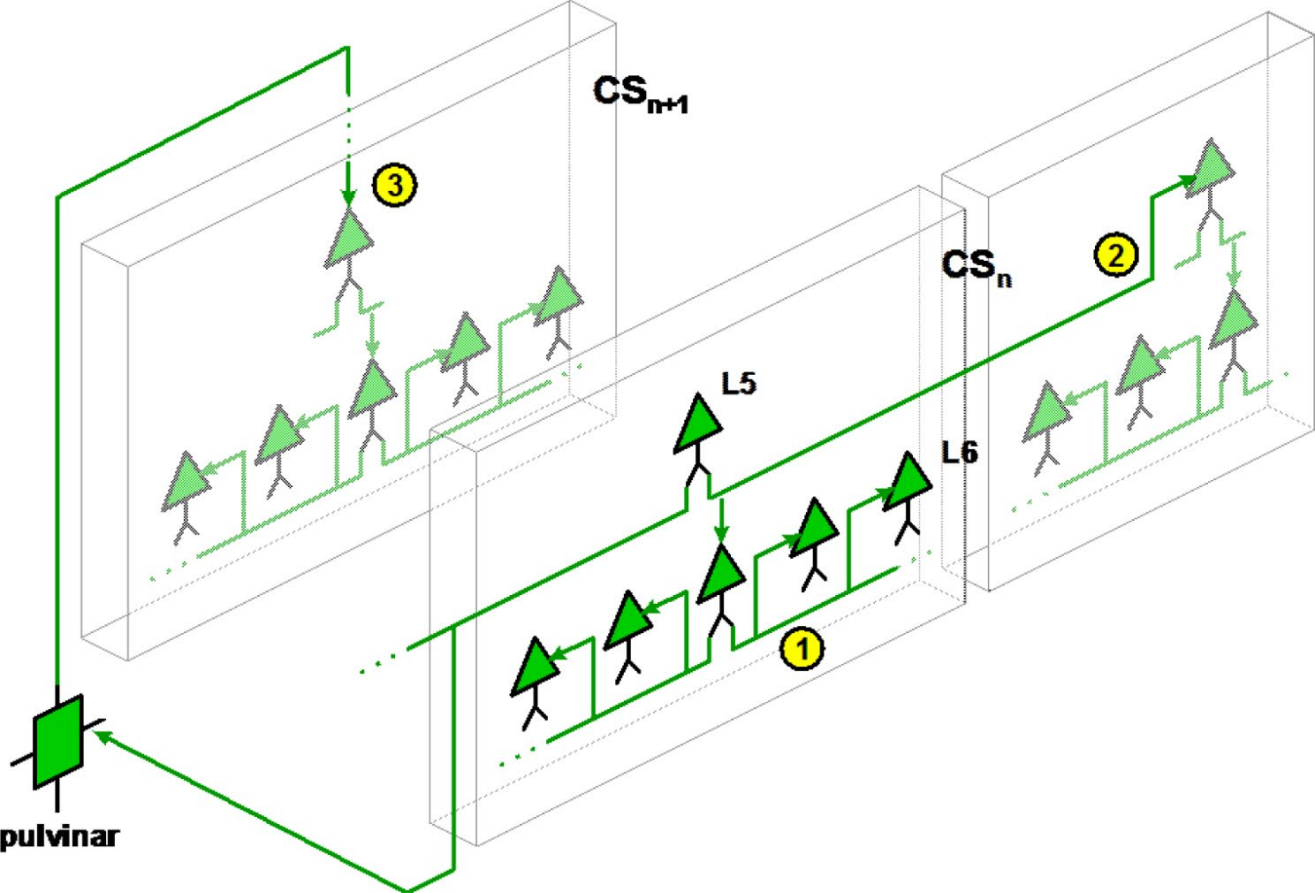
Principle of breakdown into cortical stages (CS) based on topology of projections. Two successive cortical stages are shown here: CS_n_, and CS_*n*+1_. 1: Local projections from layer 6 for local synchronization, restricted to neighboring columns within the same cortical stage. 2: Lateral projections from layer 5 for recruitment of distant localized patches, still within the same cortical stage. 3: Feedforward projections from layer 5, mediated by the pulvinar, for synchronization of localized areas pertaining to the next cortical stage

A priori, areas belonging to the same cortical stage, i.e. areas where activation starts at almost the same instant for a simple and sudden stimulus, are areas mutually connected by lateral projections (cortico-cortical projections of layer 5), whereas areas pertaining to the next cortical stage are areas where synchronization is mediated by the pulvinar, as illustrated by Fig. 6. Thus, let us consider the question from the standpoint of the pulvinar. Seven divisions have been identified for the primate pulvinar [27, 47]. There may be a link between this partitioning and the temporal progression of activation within the visual cortex.

Beside this, three time-clusters have been dissociated, centered respectively on ~ 100 ms, 125 ms and 170 ms after stimulation, based on EEG acquisitions associated with fMRI source localization [72], using a checkerboard pattern reversal condition. A similar study found the same number of clusters, using cortico-cortical evoked potentials and diffusion-weighted imaging: cluster 1 between 0 and 55 ms, cluster 2 between 55 and 107 ms, cluster 3 between 107 and 200 ms [68].

Can we consider these time-clusters as the cortical stages present between V1 and the frontal areas, or are they only part of them? This is a major question, hence the need of an experiment using EEG, or MEG recordings, to discern each cortical stage and to determine its timing. Now that we have developed this notion of cortical stage, we come back to the analysis.

### Organization of coupling between successive cortical stages

The projections linking two successive cortical stages are essential for the sequencing of the information processing. Everything is optimized within a cortical stage, as we have shown, to guarantee the best possible synchronism between all signals linked to the same visual object. We will now analyze the coupling between one cortical stage and the next, i.e. between a population of active cells belonging to one stage, carrying all the details of information relating to a visual object at a given level of abstraction, and their axonal targets at the next stage.

Due to the crumpled cortical structure and to the relative location of source and destination, the length of cortico-cortical axons reaching a same target is highly variable.

Consider for example a parietal associative area that receives supragranular projections from a neighboring area belonging to the dorsal path, and from another belonging to the ventral path. Although synchrony ensures a contained time difference between the two populations of spikes at their respective source, the difference in path length implies that spikes received from the ventral path are significantly delayed compared to those from the neighboring area. Conversely, this time difference does not affect the thalamo-cortical links, thanks to the myelination which compensates the length differences [44].

**Fig. 7:**
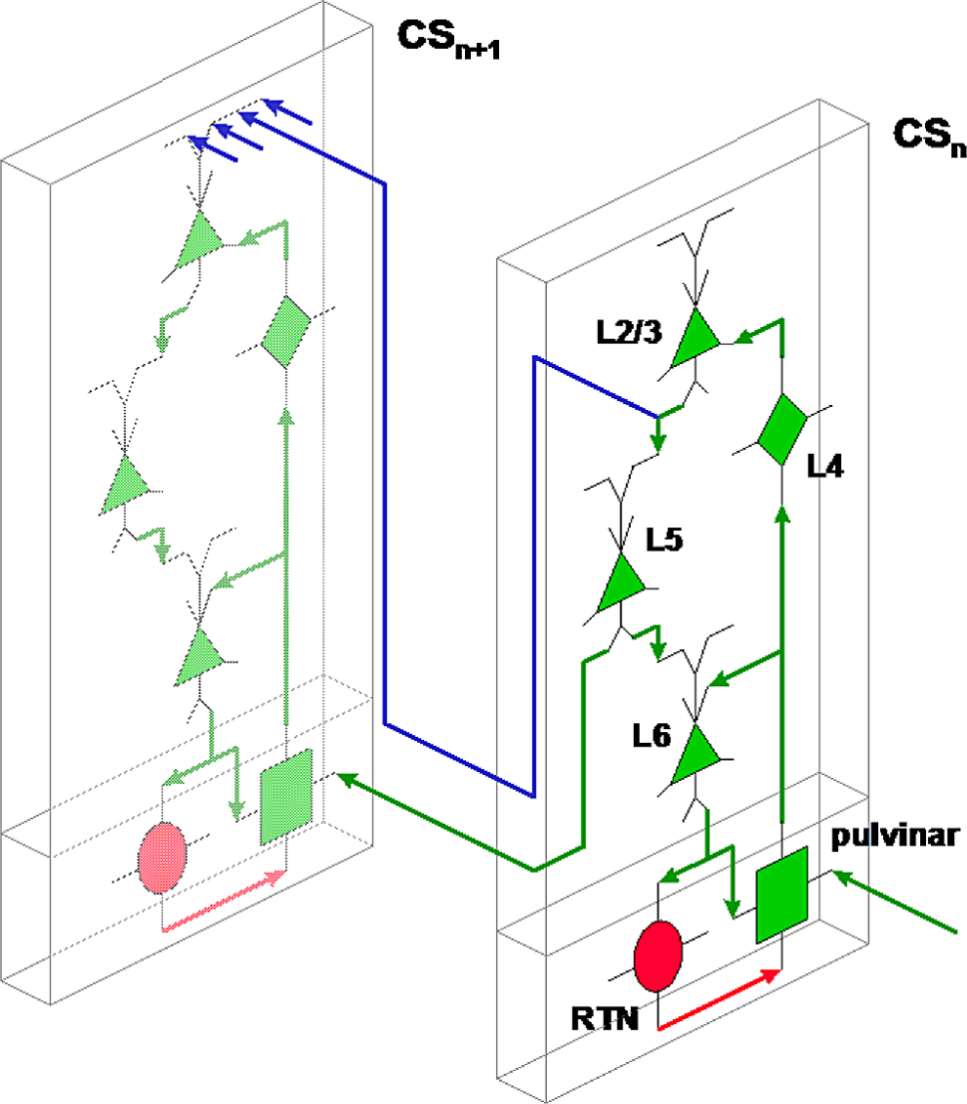
Coupling organization between successive cortical stages. Blue links: direct projections linking layers 2 and 3 to the same layers of the next cortical stage. Green link from layer 5 to the next cortical stage: low latency link for the synchronization signal

This aspect emphasizes the key role played by the time-optimized network, made up of the lower layers (4 to 6) and the pulvinar, as opposed to the cortico-cortical links, of variable lengths (Fig. 7).

### Sequence of activation within a cortical column

**Fig. 8:**
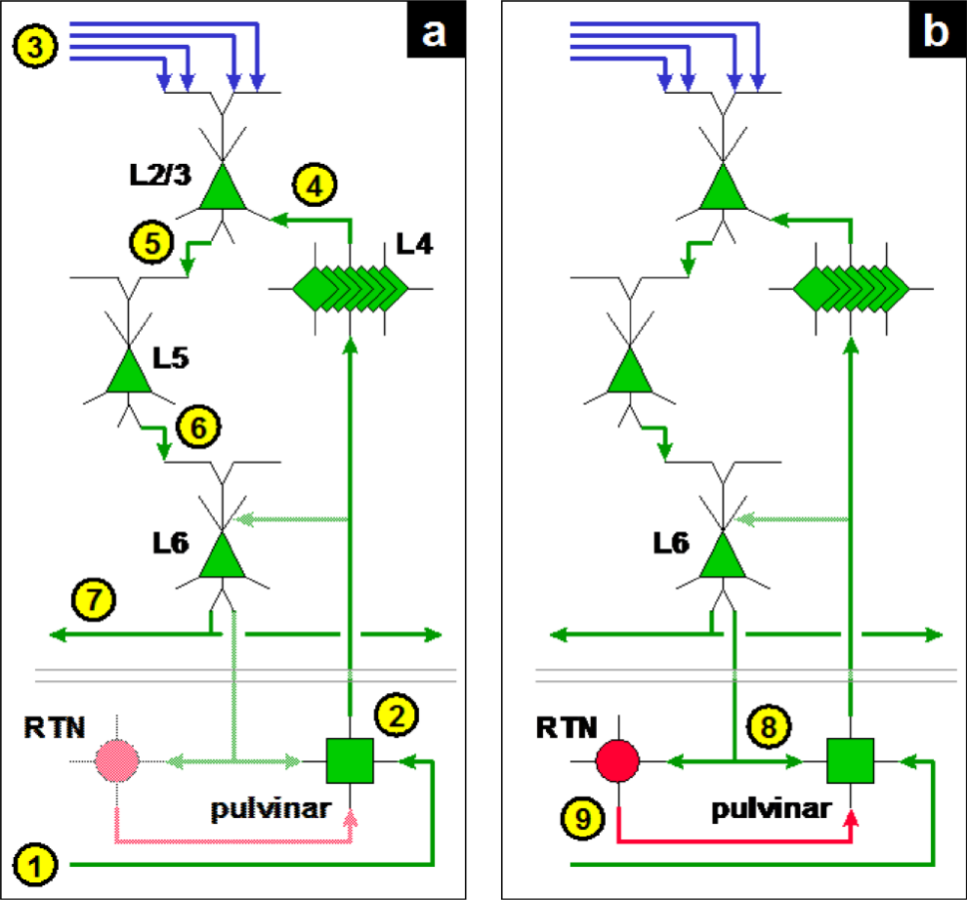
Activation sequence within a cortical minicolumn. (**a**) Reception of afferent spikes from the preceding cortical stage and sequence of activation in the cortical minicolumn. (**b**) Feedback loop through the pulvinar (excitatory) and RTN (inhibitory), leading as here explained to the spacing effect and to the synchronous excitation of adjacent minicolumns

Now that we have developed the organization of coupling between successive cortical stages, we analyze the scheme of propagation, this time along the vertical axis (cortical minicolumn and associated thalamic cells), for the feedforward stream (Fig. 8).

Numbers in yellow circles illustrate the sequence steps.

1: a set of synchronous spikes is received from the preceding cortical stage.

2: these spikes are propagated by the relay cells to layer 4 of the associated cortical minicolumn.

3: in parallel, direct projections from the preceding cortical stage are received by the supragranular cells, via their apical dendrites; the depolarization of each cell depends on the activity of its afferents.

4: supralinear summation of active afferents from the pulvinar is carried out by the stellate cell cluster (layer 4).

5: supragranular pyramidal cells receive the spikes from layer 4 near their soma, producing the firing of the most depolarized cells, with selectivity thanks to mutual inhibition between competing cells (the winner-take-all mechanism).

6: activity spreads to layer 5, with the same effect of selectivity.

7: then to layer 6; activity spreads to neighboring minicolumns via the lateral projections.

8: spikes from layer 6 reach pulvinar relay cells, with collateral innervation of the RTN; these signals affect, among others, the relay cells active in step 2; by targeting the distal endings of relay cells, these projections play a modulatory role, with the effect of reinforcing already active cells; this loop also influences neighboring relay cells: it induces the recruitment of cells related to the perception of the same visual element, allowing their synchronous activation at the next gamma cycle.

9: the RTN also receives spikes from layer 6; it produces a slightly delayed inhibitory signal, which acts as a spacer, as described before, the base of the gamma cycle.

This sequence restarts when inhibition by the RTN ceases.

### Dynamic behavior of gamma frequency

The analysis is nearly completed, as we have built a coherent view between the structural aspects, links, and sequences. We must make a small aside about the dynamic behavior of gamma cycles, as it matters on the scheme adopted for the experiment.

Before this, we need to clarify which gamma oscillations we are talking about: those related to perception hence to synchrony. Signals in the gamma band are present in V1 and V2 [37, 38, 40]; they are coded like the spikes from the ganglion cells, e.g. the higher the frequency, the higher the local visual contrast. We do not address here this type of signal, but the bursts of synchronous activations evoked by a same visual object, observed throughout the higher-order areas.

We can mention several examples regarding the behavior of these synchronization signals.

The first example concerns cats exposed to stimuli composed of drifting sinusoidal gratings [4]. For the cortical population analyzed, gamma oscillations are absent during the delay period, i.e. before the presence of the visual stimulus. As soon as the stimulus appears, gamma oscillations appear and their frequency is stable throughout the duration of exposure, at ~ 50 Hz. In this case, there is no significant frequency increase at the start of the stimulus.

Similar behavior is observed with rodents exposed to a random dot kinematogram [21], with a stable frequency at ~ 40 Hz.

In two other examples, attention is maintained on a fixation point and a sudden shift of attention is induced [20, 43]. In these two cases, the ~ 50 Hz oscillation is present and stable, both before and after the shift of attention, but a significant frequency increase on attention shift is observed in such cases.

We could have quoted other similar examples. The general rule is a stable gamma frequency when the stimulus is stable, at the attended location, and a brief increase in the observed frequency on attention shift. This observation leads us to prefer a scenario without attention shift, in the context of the present objective. Since we are analyzing time processes, anything that guarantees a simple and stable situation is preferable.

### Experiment description and results

The elements developed here before focus on the gamma cycle and its incidence on the synchronous propagation of excitations from one cortical stage to the subsequent one. On each cycle, the activation progresses from one stage to the next one. We have also mentioned that the gamma frequency is nearly deterministic in observation conditions. The consequence of these observations is that the processing delay should be as much deterministic. To assess the viability of this hypothesis, we have built up an experiment to evaluate the determinism of the global RT, i.e. the sum of the elementary delays introduced by the succession of cortical stages.

We have mentioned the travel time of the activation wave propagating from V1 to the PFC, ~ 300 ms, followed by the massive activation of frontal areas in the case of conscious perception, with a rebound of activity of the earlier activated centers [7]. Such delay was surprising. It could have been highly variable, depending on many factors, a sum of individual latencies, with wide variations around a largely fluctuating mean value. In contrast, the EEG recordings show that for the same type of stimulus and the same subject, the timing of activation is almost identical over the trials (as shown by results, refer to [7, 8, 32]. We have also pointed out the relative stability of gamma frequency, for a same subject and under similar conditions.

This double relative determinism was unexpected, considering the huge complexity of the sensory chain, and the fact that the latency of each individual cell is highly variable. It seems to confirm that within this complex organization, a specific process is present, one that could be described as quasi-mechanical, simple but with a deterministic effect.

**Fig. 9:**
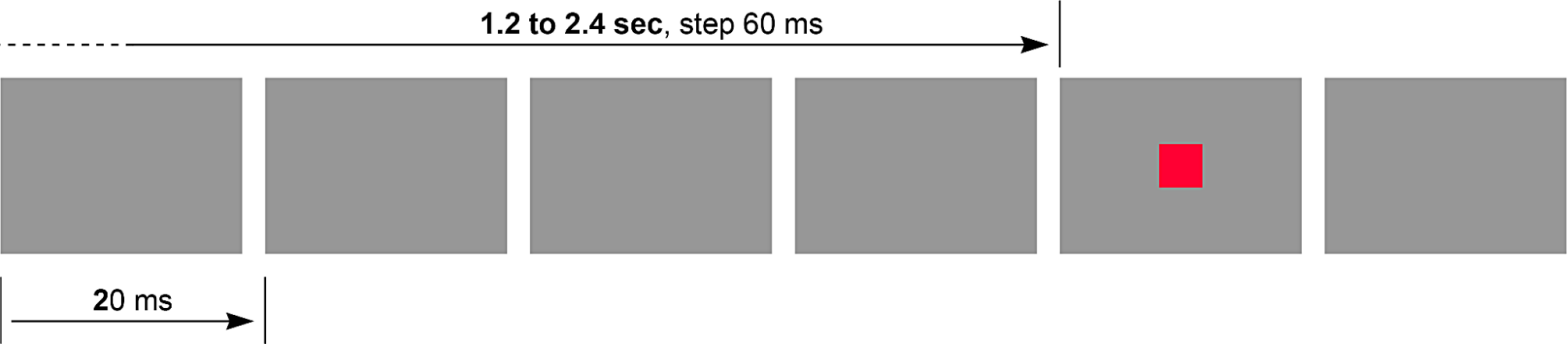
The basic scenario: zoomed-in visual stimulus, flashed after a random interval. The random target appears over a single video frame (20 ms). Random target spacing: 1.2 to 2.4 s, in steps of 3 video frames. The actual size of the random target on the screen is 2 cm^2^. Target position is fixed, centered. Background is uniform dark gray

To assess the determinism of delay, an experiment was set up, based on a bright square (2 cm^2^) displayed after a random time interval (flat random distribution, from 1.2 to 2.4 s, in 60 ms steps), the duration of which is limited to a single video frame (20 ms) on a permanent dark background (illustrated by Fig. 9). The subject strikes a key at each occurrence, and statistics are calculated for the response time.

The intention here is purely related to the assessment of the travel time, meaning that we have chosen the simplest visual stimulus, excluding any need of analysis or interpretation. The shape of the visual object does not matter, the subject has just to strike any key as soon as the target is perceived. So, this is not a processing delay: we analyze here the time needed for the wave of activation to travel from the retina up to the physical reaction. It matters to note that even with such a simple visual task and stimulus, the RT is high. There is no real visual processing, especially no segmentation (one single object, small) and no need to recognize the nature of this object. Despite this, the RT is significant, around 280 ms. This justifies the present intention to try to understand the causes of such latency, i.e. to identify the contributors to this value of RT and their incidences.

Statistics are here based on over 1,000 trials, split as 6 sessions of 5-minute, randomly distributed over 6 days. Refer to the experimental protocol (appendix) for a detailed description of the set-up and analysis process.

**Fig. 10:**
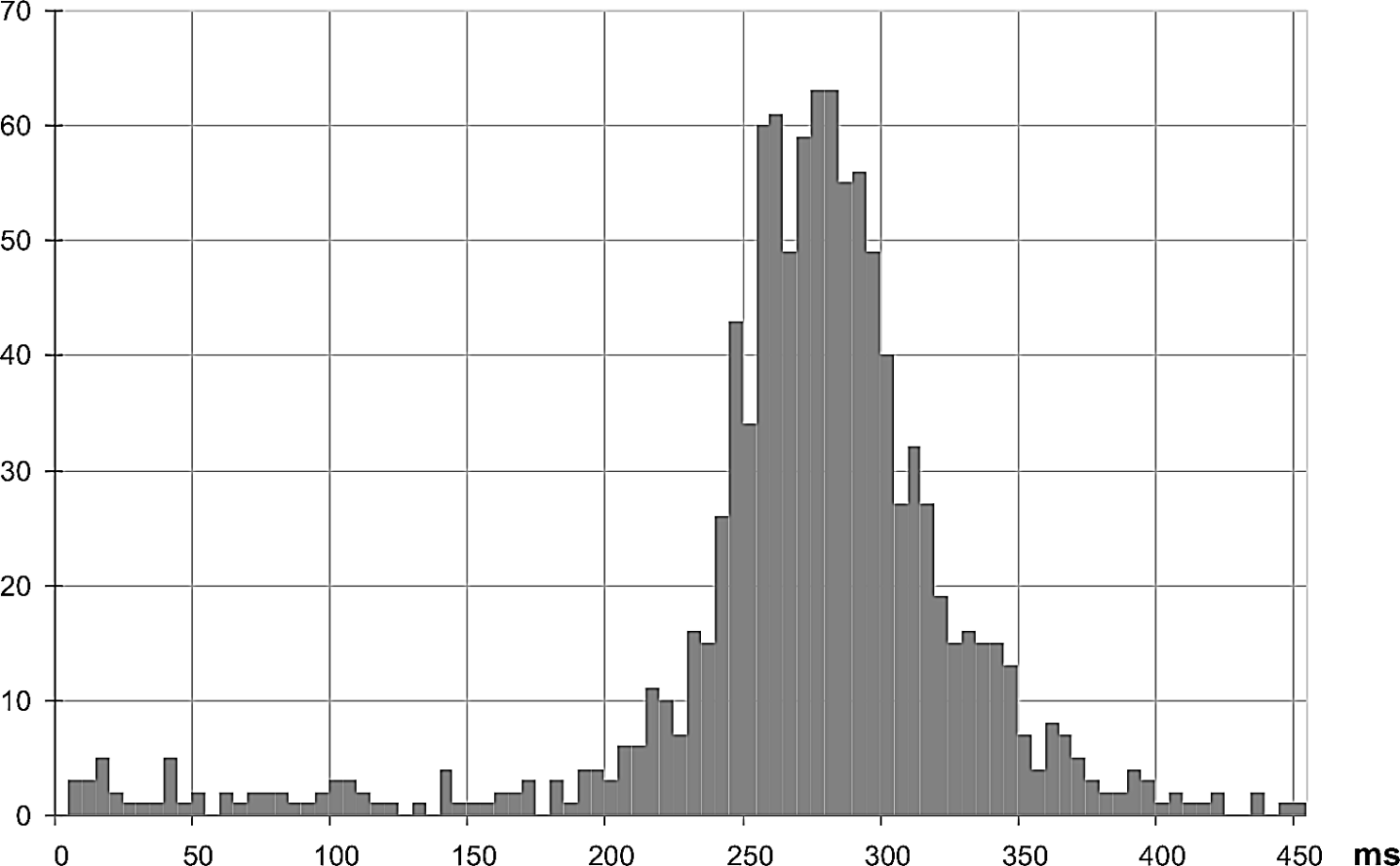
Histogram of response delay measured over 1,090 trials, with 5 ms bins. Null delays have been eluded as irrelevant, effects of erratic strikes

**Fig. 11:**
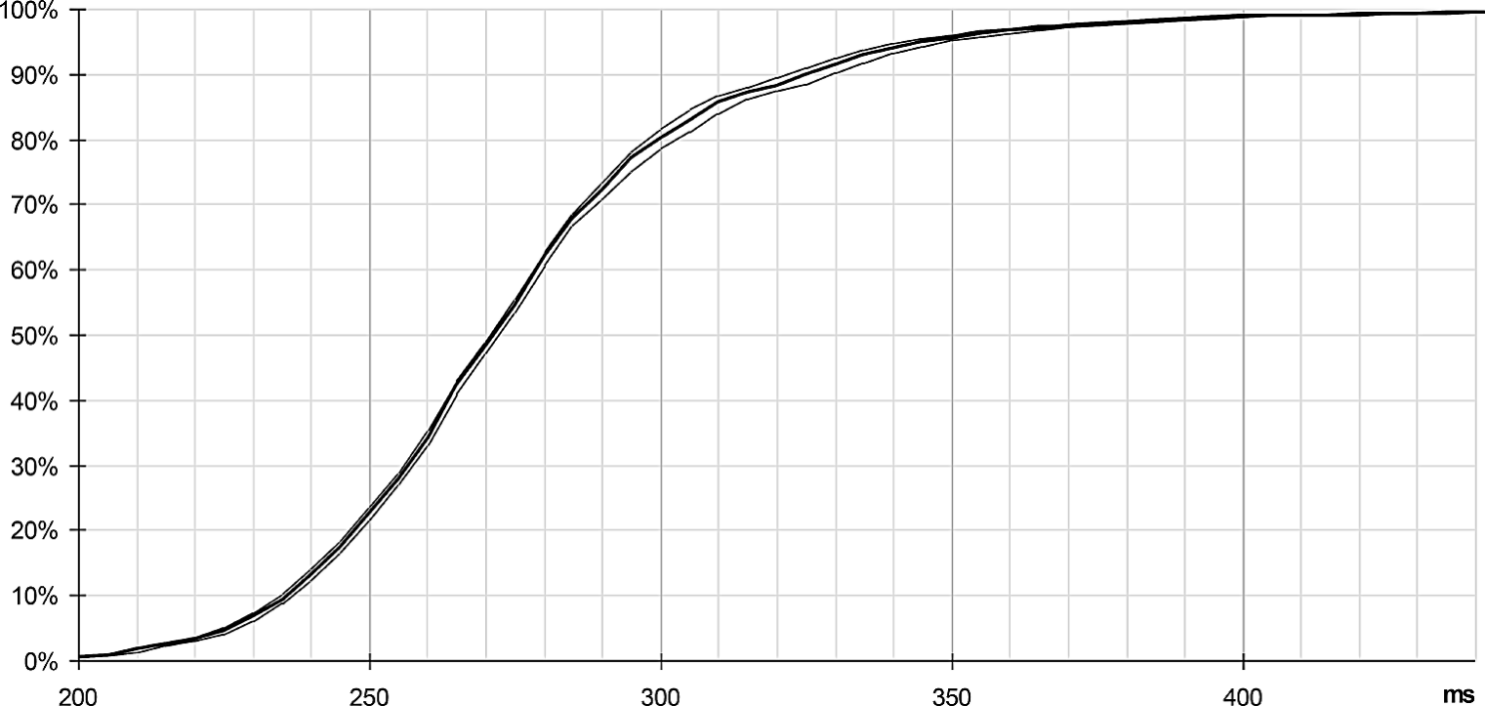
Cumulative distribution, beyond 200 ms (refer to Appendix - Experimental protocol). Statistics on 1018 valid samples (> 200 ms): mean = 281 ms, SD = 38 ms. Thin lines indicate 95% confidence intervals

The histogram obtained in such conditions clearly shows a peak between 245 and 300 ms (Fig. 10). The average response time calculated on the recorded values is 281 ms, based on 1018 trials (samples < 200 ms have been ignored, refer to appendix) (See Fig. 11).

**Fig. 12:**
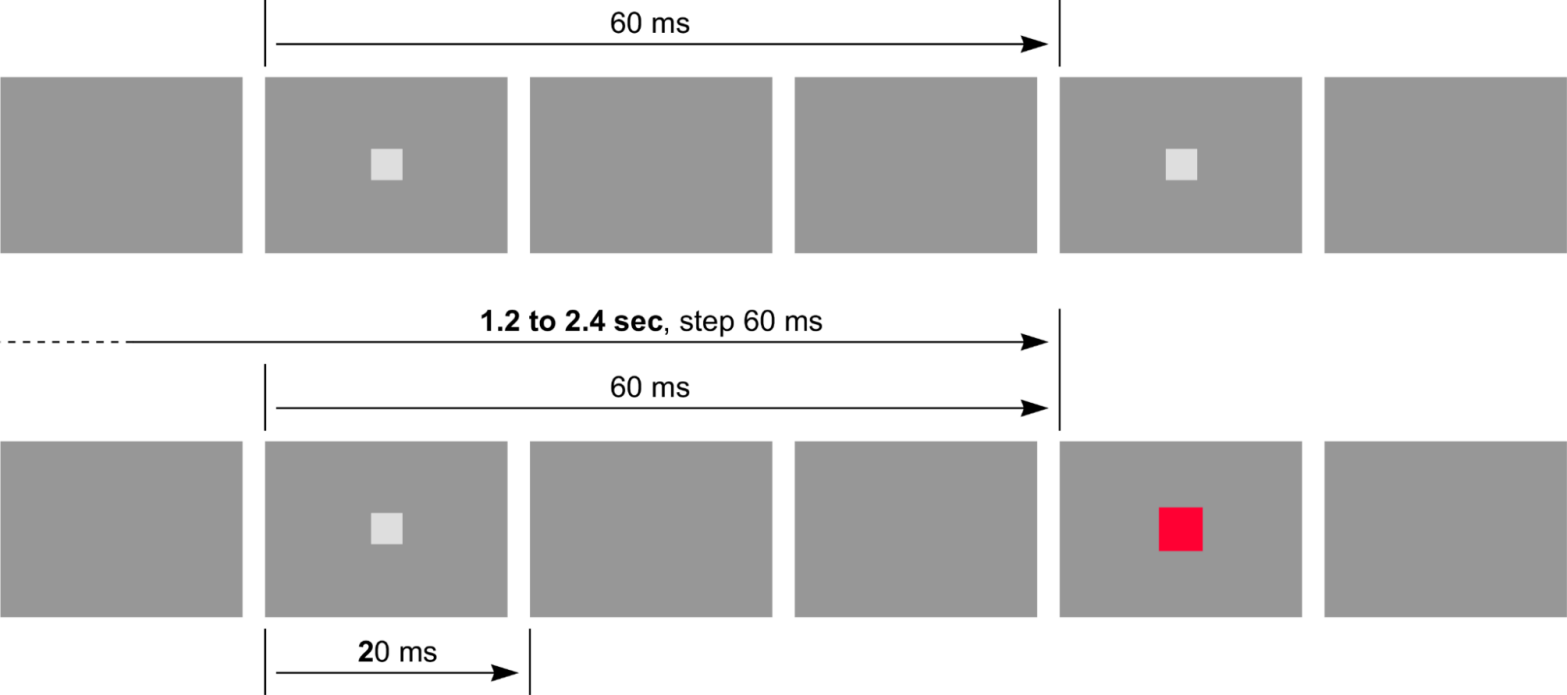
The extended scenario: a flashing cyclic target is added to the basic scenario. The period is 60 ms. The red random target is strictly in phase with the gray cyclic target. The actual size of the cyclic target on the screen is 1 cm^2^, while the size of random target is 2 cm^2^. Positioning is identical for both targets. This second scenario is strictly identical to the basic scenario, except the addition of the cyclic target

To take a closer look at synchronization mechanisms, the initial paradigm was extended, adding a cyclic flashed target, smaller than the random target (1 cm^2^, vs. 2 cm^2^ for the random target), with a different color (faded gray, whereas the random target is bright red). The main target is displayed with the same random process as in the initial experiment. The cyclic target flashes once every 3 video frames, i.e. 20 ms every 60 ms. When displayed, the main target replaces the cyclic target, ensuring that there is no phase shift in relation to the cyclic target (Fig. 12).

**Fig. 13:**
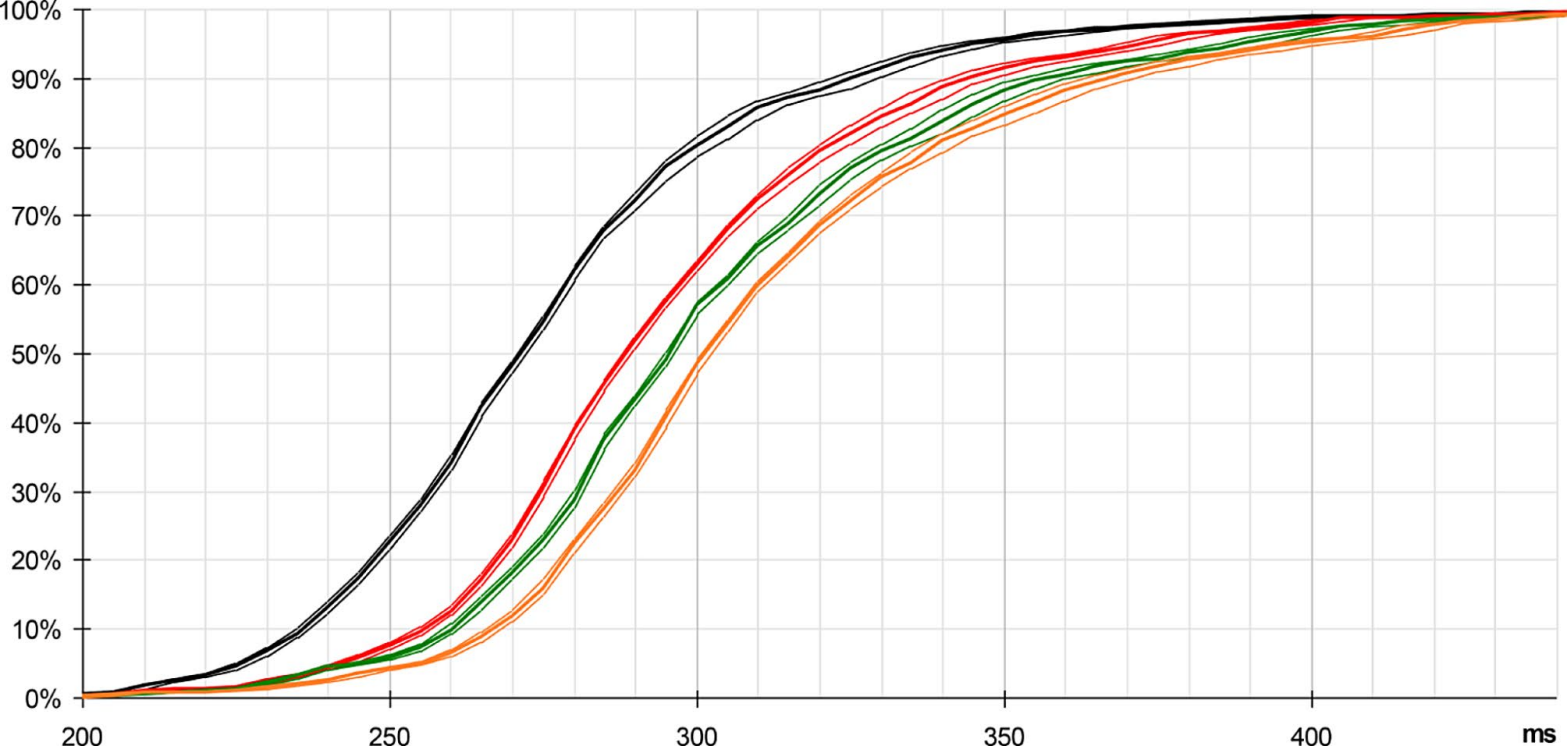
Cumulative distributions obtained with cyclic targets, compared with the initial results. Respectively without cyclic target (black plot), with cyclic target added every 60 ms (red plot), 80 (green plot) and 100 ms (orange plot). Statistics: mean = 281 ms, 301 ms, 308 ms, 315 ms, on 1018, 1161, 1089, 1025 valid samples (> 200 ms); SD = 38 ms, 39 ms, 42 ms, 43 ms. Thin lines show 95% confidence interval

Under these conditions, a time-lag appears on the cumulative distribution (Fig. 13, red curve, compared with the black curve, obtained with the initial scenario). The conditions for the random target are unchanged, allowing a direct comparison of results.

The same paradigm was repeated, this time with a period of 80 and then 100 ms for the cyclic targets. In all cases, the random target is displayed strictly in phase with the cyclic targets, meaning that the gap between random targets in these cases is extended to a multiple of 80 and 100 ms, respectively.

It should be noted that all sessions were randomly interleaved over more than one week: the baseline scenario and the scenarios with the 60, 80 and 100 ms cyclic targets. The aim of this interleaved planning was to avoid that results could be affected by altered conditions, habituation effect or a possible boredom effect: all scenarios were randomly shuffled, with the same duration. The variations observed throughout these sessions are mentioned in the experimental protocol, confirming the absence of long-term effects.

An unexpected fact concerns the time-lag observed between the four plots, which seems almost deterministic: the shift of each colored plot, respectively with a cyclic target of 60, 80 and 100 ms, versus the plot obtained with the initial scenario, seems almost linearly linked to the period of the cyclic targets, time-lag measured at 20, 27 and 34 ms respectively.

Thus, the addition of the cyclic target is not without effect on the perception of the random target, since the response appears shifted in time, the observed delay increasing with the period of these cyclic distractors. Once again, the relative determinism of this effect seems surprising in such a context.

We shall now analyze what lessons we can draw from these results, inferences which of course remain to be confirmed.

### Analysis of the stimulus-response delay and its implications

We have seen that by adopting the same scenario based on random targets, the addition of the cyclic target slightly increases the overall response time, and that this additional delay is related to the period of the cyclic target. We also mentioned that in this scenario, the random target always replaces an expected appearance of the cyclic target. There is therefore no phase shift between the random target and the cyclic target, as shown in Fig. 12.

**Fig. 14:**
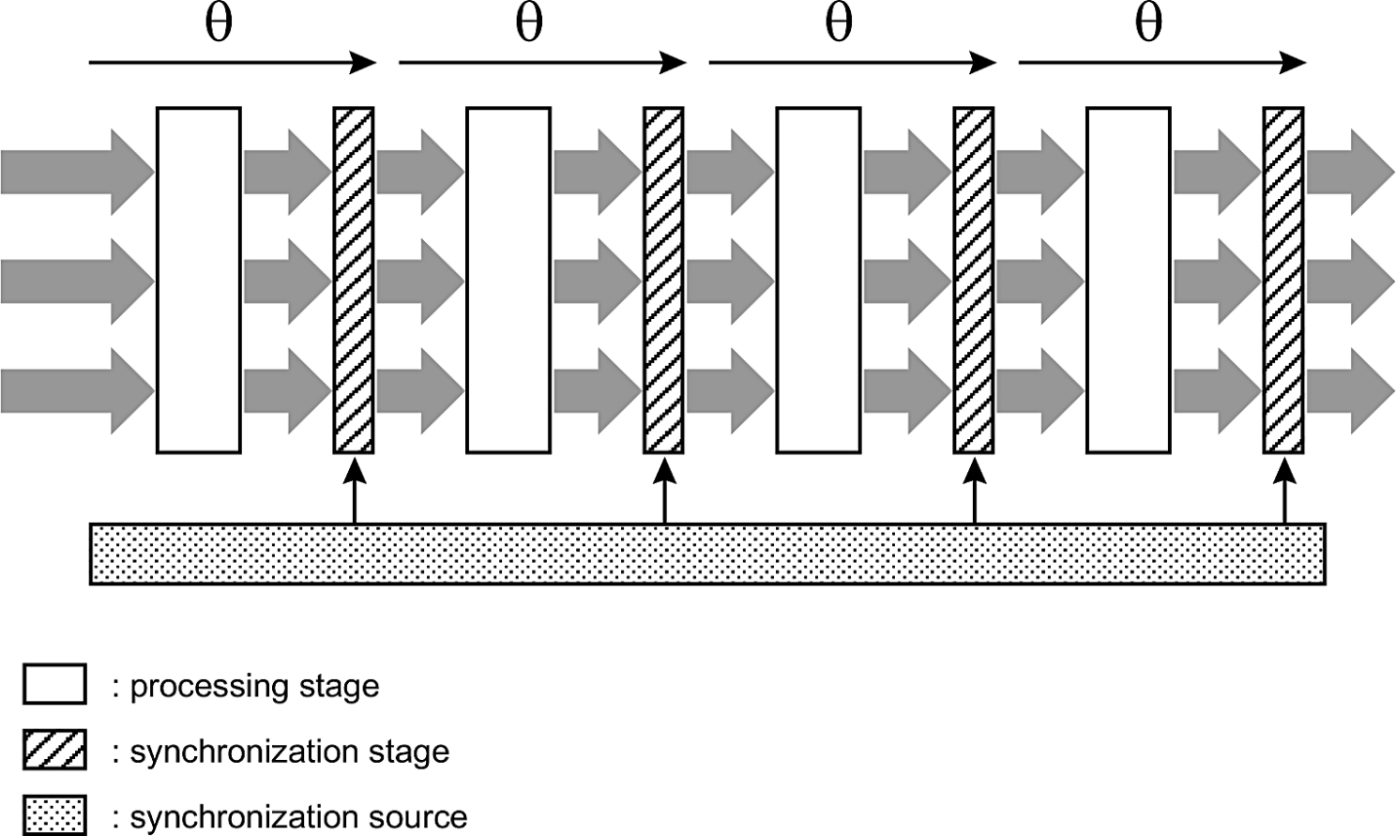
Sketch illustrating a synchronous digital processing chain, a basic structure found in electronic systems. The analogy with the cortical synchronization scheme is important, as the same type of problem is present in both cases, a purely physical problem, linked to timing constraints. Digital signals are sampled and held over the cycle of duration θ. Each processing step produces a specific operation, e.g. summation, subtraction, filtering, etc. At each cycle, the samples processed by the first stage are passed on to the second stage, and so on till the last stage. The complete running time is n x θ, where n is the number of synchronization stages

These elements may be useful in analyzing the mechanisms of perception. To do this, we need to digress for a moment and consider the example of a synchronous digital processing chain, as illustrated in Fig. 14, referred to in electronic engineering as a pipeline structure. Of course, we do not identify the cortical system with such an idealized structure: they belong to completely different worlds. The aim is simply to describe the effects produced by a succession of stages operating synchronously, something purely physical.

In such a processing chain, individual signals are transmitted in parallel from one stage to the next. The synchronization frequency, F, is generally common to the entire chain. This frequency is set in such a way that all unitary signals transmitted to the next stage are effectively stabilized before being used by that next stage: a guard time is systematically guaranteed.

In such a chain, the delay introduced by each step is: θ = 1 / F.

The total travel time is n x θ, where n is the number of synchronization stages.

This analogy, on which we propose to base ourselves, concerns a domain very far from our current subject, but it is nevertheless relevant in the sense that the underlying problem is the same in both cases, a problem related to timing constraints and synchronization. This problem is even more acute in the case of a cortical chain, where the propagation of individual signals is strongly influenced by the significant lengths of axonal projections and the diversity of these lengths, while synchronization is strategic.

We have seen in the previous developments, particularly those relating to structural and temporal aspects, that the balance between excitation and inhibition plays a critical role for each projection, that many signals are involved in parallel and that all signals relating to the same visual component must be received by the next cortical stage within a delimited time window. This is the same type of situation as for a digital processing chain, with the timing aspects constituting the core of the problem in both cases, purely physical constraints.

The only way to deal with this type of problem for a digital processing chain is to optimize synchronization, by systematically incorporating safety delays. The equivalent principle in the case of cortical mechanisms involves RTN and gamma cycles. We have described the role of RTN, which imposes a time lapse before the next gamma cycle.

The gamma cycle can therefore ensure that all signals relating to the same visual component are taken into account by the targeted cortical area at the right moment, materialized by the signals transmitted by the pulvinar to the cortical columns. It should be stressed that these signals are the most precise in time of all the projections we have detailed.

The pulvinar plays a coordinating role [42, 45]. The term coordinator seems appropriate, as the pulvinar synchronizes numerous cortical assemblies that operate in parallel, for the simultaneous processing of the observed visual object and its visual environment. This is not something intentional, intelligent or anything else; this is a mechanical function, for which we have detailed the principle of dependencies. Other functions are attached to the pulvinar, notably everything relating to attention. Here, we consider one specific aspect of the pulvinar, its important role as cortical coordinator in the sensory context.

In the case of a digital processing chain, the propagation of a set of signals takes place in a single step, a cycle at synchronization frequency F. When a set of signals is present at the input of the chain, it is processed by the first stage, then propagated to the second stage at the next synchronization cycle, and so on from stage to stage, at each cycle.

Conversely, in the cortical system, propagation is progressive. A first population of neurons is activated, and then, through a recruitment effect, at the next cycle, other sets join the initial population, and so on over a number of cycles that depends on the complexity of the object to be isolated within the visual scene. Both lateral and feedback projections contribute significantly to this recruitment effect. Response time is therefore highly dependent on the nature of the visual object. For example, the detection of an animal in a visual scene is faster than that of a man-made object such as a vehicle [6], a notable case that would be interesting to investigate in future steps, using the method described hereafter.

For these reasons, we insisted on an experiment based on a very simple visual object. With such a visual stimulus, lateral recruitment is still required, but the activation process of the cortical areas involved in perception is faster and more deterministic than for most visual objects. Moreover, object recognition is not necessary in this case, as the shape of the target (a simple square) is fixed and non-significant. Here, this is essentially a feed-forward processing.

We noted that here, the response time histogram (Fig. 10) is rather narrow. This histogram can be related to the moderate fluctuations of gamma frequency with such visual scenario.

This may indicate a direct link between these two parameters, similarly to a digital processing chain, although the comparison does not go beyond this observation.

If confirmed, this indication might mean that the delay introduced by the cortical chain would be N times the gamma period for a given visual object, like the effect described for a synchronous processing chain, although here the activation is progressive, i.e. not instantaneous. N would be the number of cortical stages as defined here, i.e. the number of stages whose activity starts at the same instant following the occurrence of the visual stimulus. This assumption remains to be validated.

On this same purpose, we can mention the results produced by Schmolesky et al. [46] showing the average delay introduced at successive stages of the visual sensory chain (in this case, V1 to V2, V2 to V4) on occurrence of visual flash, is close to the gamma period. This is not the case for the areas fed by the extragenicular pathway, as commented later.

So, a possible approach to go forward in the present investigation would be to repeat the experimental protocol based on flashed targets, this time with the addition of EEG recordings and high-resolution analysis, as defined by B. Burle [3]. If the stimulus duration of one video frame produces a signal too weak for reliable timing analysis, the stimulus can be maintained over 2 or 3 video frames.

Evaluation of the gamma frequency and its variations over each session is necessary, for example by holding the target for a slightly longer time, every minute.

### V1 and LGN

**Fig. 15:**
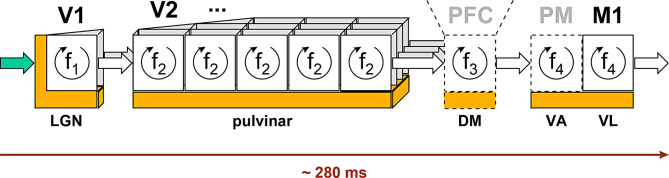
The complete chain, from LGN up to the primary motor area. This diagram illustrates the successive cortical stages and their frequency of synchronization, for the perception of an object present in the visual scene

The whole sensory chain is schematized in Fig. 15. This diagram symbolizes the dissociation process that starts in area V1 and continues throughout the cortical chain.

A side effect observed here is that the addition of cyclic target has led to a slight increase in response time, about one third of the period of this cyclic target (Fig. 13): 20 ms, 27 ms and 34 ms respectively for 60, 80 and 100 ms cyclic targets (the third of period is 20, 26.66 and 33.33 ms, respectively).

This additional delay may not be introduced at each stage of the sensory chain for a simple reason: any additional delay would in this case be multiplied by N, the number of cortical stages, for the reasons developed above (total delay = N x unitary delay), whereas here we observe a submultiple rather than a multiple effect. One possibility is that this slight additional delay be induced by an extra processing time at the first cortical stage, V1 associated with LGN. The LGN, like the MGN (Medial Geniculate Nucleus) for audition, are nuclei that differ significantly from other thalamic structures.

It is conceivable that the frequency of synchronization may differ between the sensory chain, associated with the pulvinar, and the first stage, V1, associated with the LGN, for the reasons just mentioned.

As previously developed, these frequencies, in the gamma band, might serve as a synchronization base for each stage of the cortical chain. This is also the case of frequencies f_3_ and f_4_ mentioned in this diagram, respectively for the PFC and for the motor areas: there is no reason to consider that they are identical to f_2_.

Conversely, f_2_ frequency would be identical throughout the sensory chain for a given visual object, which is consistent with the observed facts: localized evoked potentials are phase-locked to the visual stimulus [7, 11, 12, 56].

Regarding f_1_, it seems relevant to refer to interesting results obtained by Fakche and Dugué [14] who have analyzed the perceptual cycles induced with a flickering stimulus, a disk whose luminance oscillates at a frequency set between 4 and 10 Hz. A low contrast target is added. Results show that the probability of detection of this target depends on the instant of its occurrence within the inducer period. Furthermore, EEG recordings show a clear modulation of cortical activity, matching the frequency of the inducer. This activity is localized, precisely at the occipital cortex, [14], hence at the level of V1.

In a similar way, although we use a very simple visual stimulus, the top-down interactions seem to influence our results precisely when the cyclic targets are interleaved with the random target. A rapid focus on this aspect is necessary.

### The effects of top-down interactions

A key aspect of the experiment commented here is the type of visual stimulus: a simple bright figure popping up pseudo-randomly over a dark background. No mask in this case, no distractor, just a simple visual element, free of any ambiguity, thus avoiding any detailing of the target. With such stimulus, the progression is straightforward, almost flawless, but can we really exclude any form of top-down interaction?

Interesting publications address the field of top-down interactions, among them: [11, 14, 15, 24, 25, 29, 52–54, 65, 67]

The first type of top-down interaction is the feedback from higher cortical stages, via direct cortico-cortical projections. They actively contribute to the analysis of the visual scene. Through a re-entrant interaction, they facilitate the activation of the targeted cells, allowing the emergence of coherent assemblies of active neurons. So, this first mechanism is based on a facilitating influence, lowering the spiking threshold of the targeted cells.

The stimulus used for the experiment does not need any kind of interpretation. Other scenarios may require a specific reaction of the subject, according for example to the color, shape, or type of the displayed object. Here, this is not the case. The shape is constant, a square; the color is fixed, bright red, over a permanent dark background. So, there is no need of iteration in the processing course of the visual stimulus, there is no ambiguity to be resolved. There is only a wave of activation that progresses through the sensory cortex. Activation would be sustained if the visual stimulus was persistent, but this is not the case here as the stimulus is just flashed, over one single video frame (20 ms). The feedback interactions are probably present even in such simple case, but the leading wave of activation, on which depends the subject reaction, does not seem to depend on such type of influence.

This is not the case for the second type of top-down interaction, related to attention. Instead of direct cortico-cortical coupling, this second mechanism involves the thalamic circuitry. This process is not facilitating. It is instead based on inhibition, but this question goes beyond the present work (see for instance Maunsell, [34].

We have noted the additional delays observed with the cyclic targets. These targets are similar to the random target used for the RT evaluation, but they flash periodically, at a rate within or close to the alpha band, here at 10, 12.5–16.66 Hz. The scheme applied is such that the random target exactly replaces one cyclic target. The original intention was to reduce the response time by optimizing the sampling rate. The opposite effect is observed: response time is increased, with additional values of 34, 27 and 20 ms respectively.

It seems that this delay is linked to attention mechanisms. We will restrain the description to the present situation. When two or more objects are present in the observed scene, alpha oscillations (8–13 Hz) rhythmically modulate perception. Unattended objects are filtered out by the inhibition process based on alpha oscillations. Assemblies of neurons are alternatively inhibited, allowing the concurrent perception of each object. Gamma spikes are alternatively enabled for each object. In the case of two competing objects, one object is ignored while the other is effectively perceived. We can give an example of such effect, a situation we have all noticed one day. When a building is under construction, it is generally protected by a fence made of vertical wooden planks, with a small space between them. If we are static, we just see the planks. As we walk by, we can see rather clearly the scene behind the fence. This capacity results from the cyclical sampling at the optimal instants, like with an old cinematograph.

In the present case, the distractor is the cyclic target, and the attended object is the random target. The additional delay in response time might be due to the inhibition process. The fact that this delay increases with the period of the cyclic target might indicate that the frequency of the alpha oscillation is influenced by the frequency of the cyclic targets, and therefore that these targets play the role of inducer. This aspect is important, because this way of manipulating the attention cycles could be useful for further investigations of the perception mechanisms.

### Generic organization of signal propagation

**Fig. 16:**
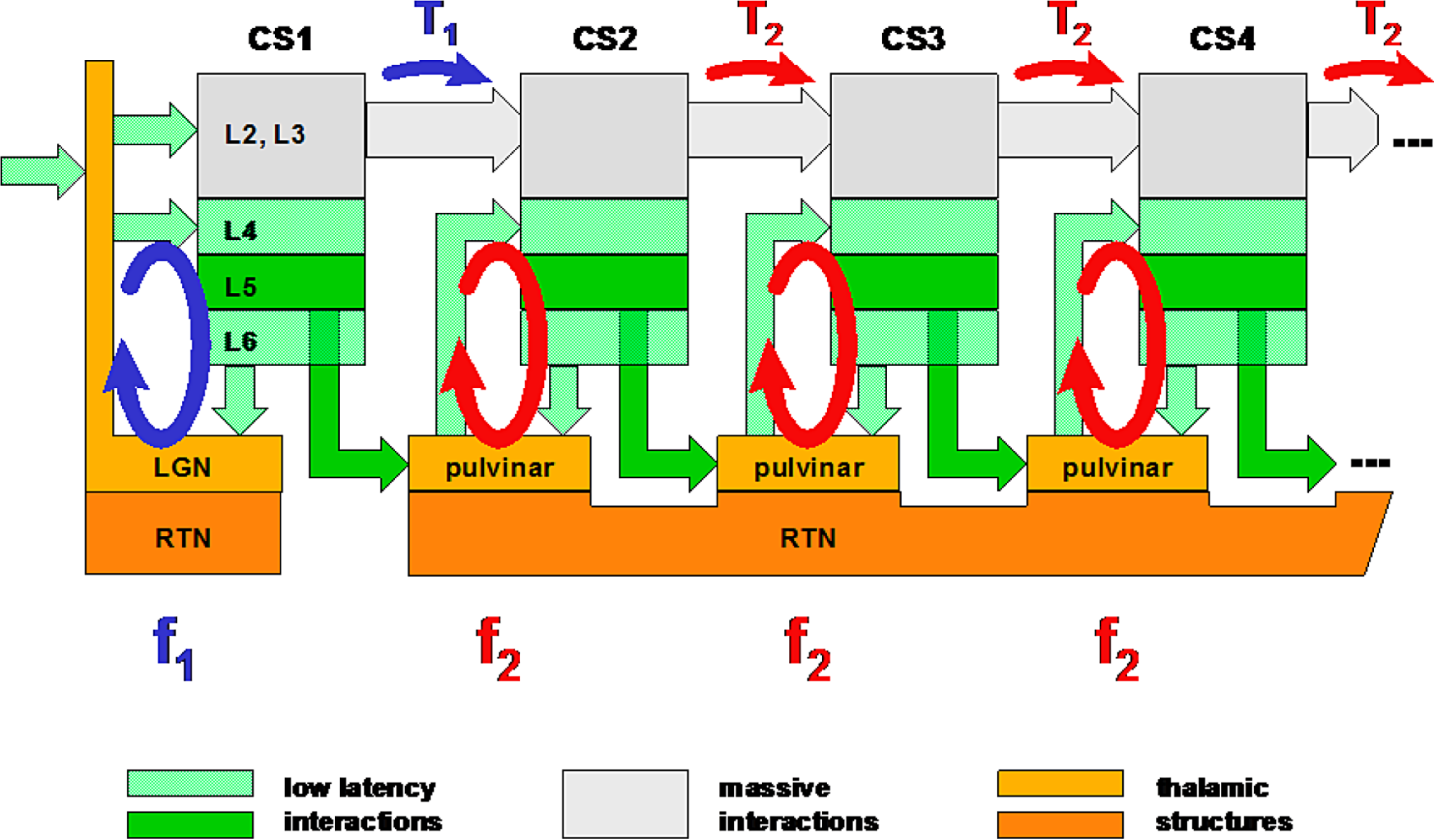
Generic organization of signal propagation. This diagram illustrates the fact that the first cortical stage (V1) operates at a specific frequency, f_1_, for the reasons detailed here, a frequency linked to the visual stimulus observed, while higher-order areas operate at a different frequency, f_2_, common to all cortical stages for a given visual object. This diagram also illustrates the particularity of the first cortical stage, where afferent signals transit via the LGN, while massive projections beyond V1 progress directly to the next cortical stage. According to the proposed analysis, pulvinar relay cells guarantee the synchrony of L5 projections prior to their application to the associated cortical columns, making a major contribution to the overall synchronization pattern

Based on these considerations, we can now focus on the first cortical stages and thus build a generic scheme (Fig. 16).

Projections from layer 5 (dark green arrows) carry the fast, time-accurate synchronization signals associated with the direct projections from the supragranular layers (gray arrows).

In line with previous comments, T_1_ delay introduced by the first cortical stage seems related to the nature of the visual stimulus (here: without, or with cyclic targets). Conversely, T_2_ delay is constant, related to the stable gamma frequency f_2_: T_2_ = 1 / f_2_.

For a given visual stimulus, the overall delay is constant, varying only with the moderate fluctuations of each gamma frequency, a situation that corresponds to the phase-locked evoked potentials [7, 11, 12, 56].

### The extrageniculate pathway

Throughout this paper, we have considered visual paths as a succession of cortical stages, where a visual stimulus elicits a wave of synchronous activity, which propagates step by step. To analyze the underlying mechanisms, we insisted on the choice of a simple visual stimulus to produce a deterministic behavior.

In contrast, the visual situations of everyday life do not follow such simple dynamics. They involve a parallel pathway that reach the area MT (Middle Temporal area) directly via the superior colliculus and pulvinar [1, 2, 27, 41, 47]. This direct pathway offers the capacity to quickly shift the attention to any potentially critical element appearing in the visual periphery.

With the onset of a visual stimulus involving a shift in attention, cortical dynamics are completely affected, due to the shortcuts mentioned. While area MT is located midway along the visual sensory chain identified by Van Essen [63], it can be activated nearly at the same instant as V1 if the superior colliculus-mediated pathway is involved (such case is illustrated in Schmolesky et al., [46]. This aspect underlines the influence of these alternative circuits.

Analysis of sensory circuits and mechanisms must therefore avoid such situation, by opting for a fixed stimulus position, adopting a dark, uniform background, and avoiding any possible cause of distraction, even non-visual ones.

## Discussion

To assess the mechanisms of sensory synchronization and the role of pulvinar in this matter, two complementary approaches have been followed here. The first is based on a review of the literature related to pulvinar-cortex interactions, the second is related to the results obtained with a simple experiment. The first was carried out starting from the elementary structural level, the second considered behavior at the global level, and we have shown that both approaches lead to a convergent point of view.

The literature review addressed the components involved in thalamo-cortical interactions and the production of gamma cycles. From these elements, we have built a model illustrating the sequence of events related to the gamma cycle. Finally, using this model, we have analyzed the propagation of activation over the sensory chain, up to a physical reaction via the motor areas.

The central aspect of cortical progression is synchrony, first locally for events related to the same visual element, then laterally as several cortical areas get simultaneously active for the same object. Perception of a given visual object in a complex scene is the result of this synchrony, which federates the different areas spread over both pathways, ventral and dorsal, despite the distance between them. Without this principle of synchronization, the signals relating to all objects present in the visual scene would be totally mixed up, and no effective perception would be possible.

Exchanges are based on two complementary networks. For the higher-order visual areas, the first array is made up of direct, massive cortico-cortical projections. The second network connects the same sources and destinations, but transits via the pulvinar. This second network is time-optimized, with latencies contained despite the variability in projection lengths [44]. The pulvinar occupies a central position, with tight temporal coupling to the associated cortical columns, enabling it to play a federative role in the synchronization process. It acts like the conductor of a symphony orchestra, enabling all its members to play exactly in phase.

We were intrigued by the delay observed between the occurrence of a visual stimulus and the massive activation of frontal areas in the case of conscious perception, ~ 300 ms [7, 8, 48]. A rather deterministic value in such context is surprising, considering the high variability of latency introduced by each neuron. The model defined here for the mechanisms of sequencing helps to analyze the causes of this determinism. The fact that the gamma cycle is also deterministic in observation conditions, ~ 40 Hz [11, 19, 55, 56] seems to confirm the relevance of the proposed model, a succession of discrete stages where each step introduces a deterministic delay related to the gamma period.

We have set up an experiment to start assessing the validity of this model, through the statistics of response time to a simple bright figure flashed over a dark uniform background. We also observe a rather sharp histogram, centered at ~ 280 ms. We have noted that we can influence this global delay by the addition of cyclic targets interleaved with the random target. Based on the collected elements, we propose that this additional delay is limited to the very first stage, V1 associated to the LGN, through the alpha oscillations related to attention mechanisms.

As some of this work is the result of interpretation and cross-checking, further work is needed to validate the proposed elements. The paradigm described here will be useful, this time with EEG or MEG recordings. The size of the random target can be increased to obtain more contrasted responses, as can the target duration. With such a visual stimulus, it will be possible to discern each node of activity in the cortical space and to evaluate the delay introduced by each stage.

Here, we used a single type of target, a square, thus avoiding the need to analyze its shape to react appropriately. It would be useful to progressively use more complex targets, in order to involve other cortical areas, in particular temporal and frontal areas.

By involving different subjects, healthy but at different ages, it will be possible to construct a map of active zones, as well as to build timing statistics according to age, for example. This will provide a set of references, helpful for the comparative analysis of cases of sensory deficiencies.

## Appendix - experimental protocol

The time measurements must be done with the best possible accuracy, ideally 1 ms.

The configuration used to obtain the present results consists of a high-end desktop computer, gamer type, ensuring the best possible reactivity. This computer is equipped with a wired keyboard. Wireless keyboards should be avoided, as some models can introduce erratic latencies. Network connections were disabled during testing, and unnecessary applications were systematically closed, to reduce software interruptions.

All possible distracting elements are avoided, even auditory elements.

The video monitor is set for comfortable viewing conditions, with medium contrast. Ambient light is dimmed. Viewing distance 55 cm.

Target duration = one video frame. The video frequency was set at 50 Hz. A comparison was made with trials at 60 Hz; the results were somewhat less accurate, probably because the targets displayed only 16.6 ms produced a weaker visual perception.

A simple software was developed for this experiment, displaying a dark permanent background (RGB = 160, 160, 160), 40 cd/m^2^, and the targets:

- Cyclic target, centered square, light gray (RGB = 210, 210, 210), 75 cd/m^2^, viewing angle ~ 1°.
- Random target, centered square, red (RGB = 255, 0, 0), 90 cd/m^2^, viewing angle ~ 1. 4°.

The target cycle can be set to any value, multiple of the 20 ms video frame. It must be noted that no synchronization of the visual stimulus onto the video frames was possible due to the software platform used for these experiments. This situation produces a random jitter of one video frame, 20 ms, resulting in a spreading of the histograms peak, non-negligible as it is 1/3 of the peak width at 50% (~ 60 ms, Fig. 10). Hence, the reality is even better than shown here, the real RT variations are a little bit more restrained.

For the baseline scenario, the cyclical targets are masked. For the extended scenarios, the cyclical targets are enabled, with a selectable period: 60, 80, 100 or 120 ms.

The random targets are spaced by an equiprobable gap between 1.2 and 2.4 s, multiple of the selected cyclic target period, and multiple of 60 ms without cyclic targets. The same timing is applied for the random target that replaces the cyclic target, strictly in phase with the cyclic targets.

The response time is measured by a click on the keyboard. The response time is recorded, as well as the gap from the previous random target.

The software application is short and simple, to guarantee the best possible timing accuracy. Statistics are evaluated using a separate and dedicated tool.

**Fig. 17:**
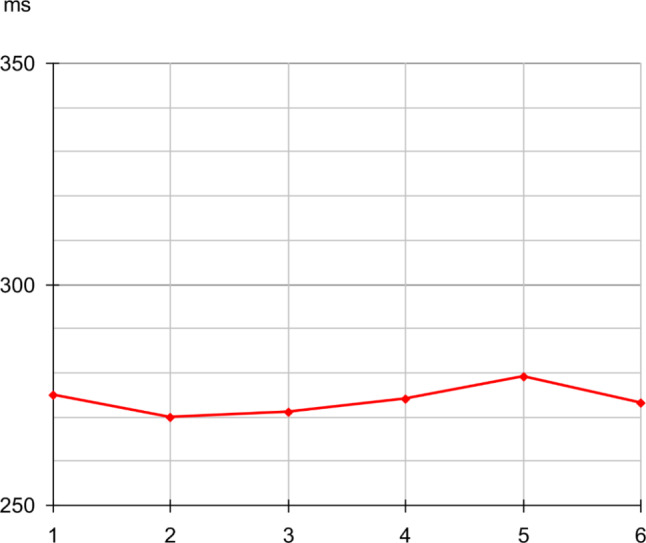
Variations of the measured average response time. Mean value compared over 6 sessions, with the same scenario, here the basic scenario (before < 200 ms screening)

All scenarios were randomly interleaved over more than one week: the baseline scenario, and the extended scenarios, alternating the 4 selectable periods. Figure 17 shows the variations of the average response time over 6 sessions, for the baseline scenario. The variations are similar for the other scenarios, with no correlation effect in these fluctuations, which means that throughout these trials, no habituation effect for example was observed. The duration of each session was 5 min. This duration was initially determined to ensure that there was no degradation of results during the session, due to a progressive loss of attention.

**Fig. 18:**
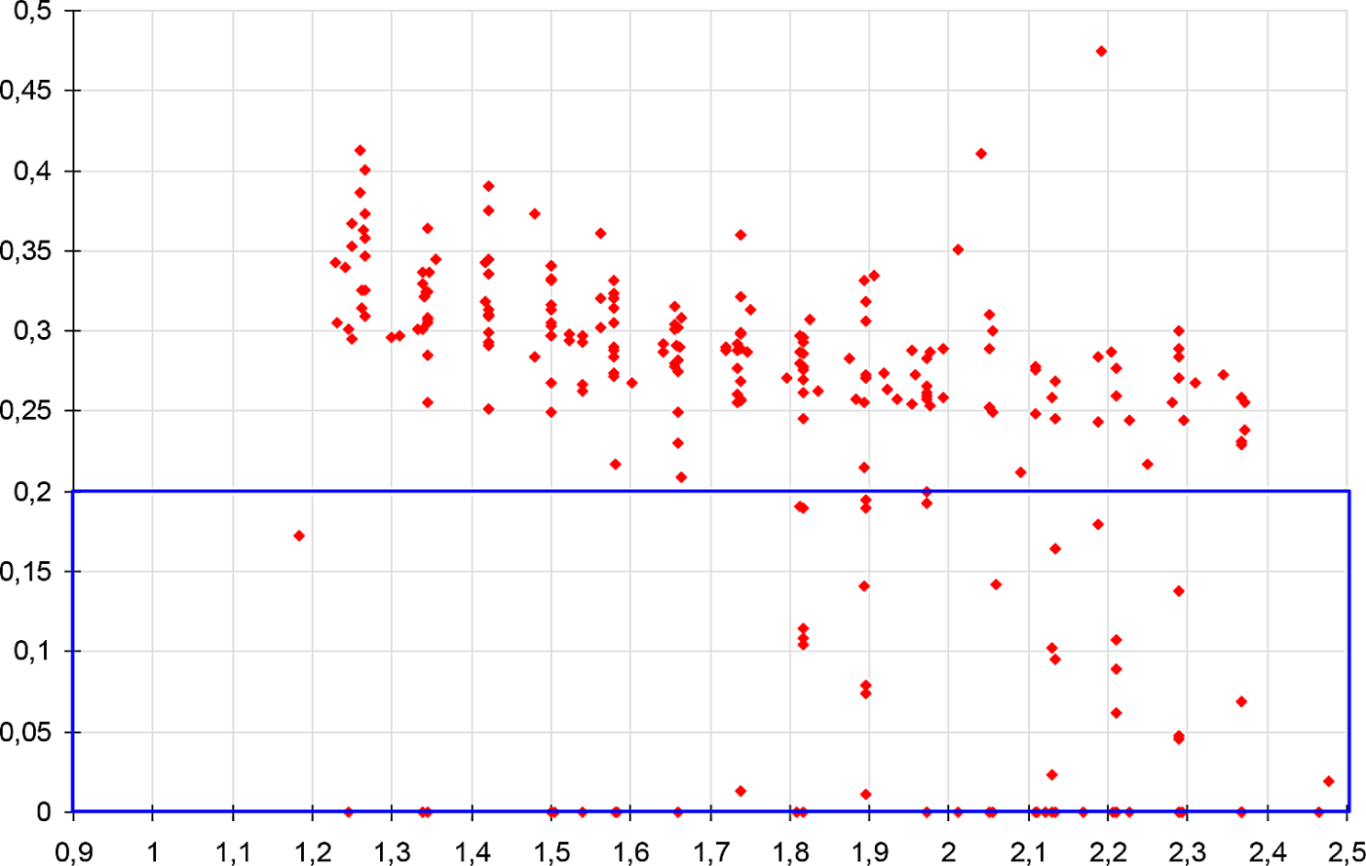
Response time vs. interval between random targets. Both axis in seconds

The interval between random targets was optimized. Too short a gap leads to automated responses, too long a gap to a gradual effect of degraded attention. Nevertheless, Fig. 18 shows a residual bias: longer intervals result in a shorter response time, and vice versa, over a noticeable range, close to 100 ms for the extreme cases, considering the average values. This effect cannot be avoided; it is even more important if a smaller range of random intervals is adopted. The range of intervals adopted, from 1.2 to 2.4 s, is a compromise without significant impact, since the main objective is to compare the results obtained with the four scenarios, all of which are affected by the same bias.

Figure 18 shows that many samples stand below 200 ms, range outlined by the blue rectangle. These samples were not considered in the cumulated distributions, as they are considered purely erratic, unrelated to an actual perception of the target, just erroneous clicks, some of them even before the random target appeared. No other filters were applied. The statistics for each scenario were performed with more than 1000 samples after this screening.

**Fig. 19:**
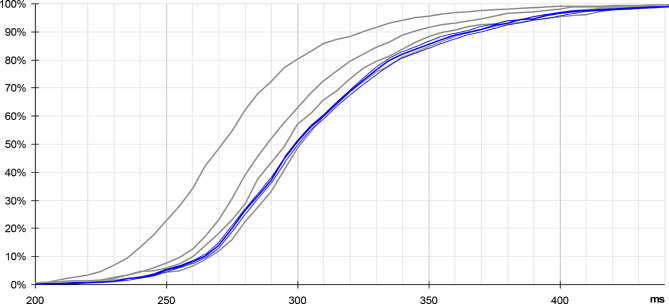
Cumulative distribution with 120 ms cyclical targets. Refer to blue curve, compared with results presented in Fig. 13. Statistics: mean = 313 ms, on 1053 valid samples (> 200 ms); SD = 42 ms. Thin blue lines show 95% confidence interval

An additional scenario was interleaved with the other scenarios, with a period of 120 ms. The results are reported in Fig. 19, blue curve. We note in this case that the monotonous progression obtained with the 60–100 ms scenarios (thin gray curves) is not maintained. Although based on the same number of samples, this additional plot is less regular than the first 4 plots. A possible reason would be that the effect of locking onto the cyclic targets features a limited range. This fifth curve is relatively unstable, while the first four plots are quite similar, just affected by an offset.

This might be considered as further evidence that the LGN + V1 pair introduces the slight additional delay when comparing the results obtained with the different scenarios, with a less accurate locking effect when the frequency exceeds a given ratio between the gamma rhythm and cyclic targets. Analysis of EEG-acquired evoked signals in the occipital area might confirm, or not, this hypothesis. Trials with even longer periods of cyclical targets show the same type of effect.

This experimentation can be easily reproduced. The results provided here have all been obtained by the first author himself, with normal vision, age > 60.

The set-up can be improved by using a simple micro-computer with minimized operating system, and LEDs instead of a video monitor to suppress the lag due to the video frequency. Time accuracy for the measurement of the response time could be better than 1 ms with such a platform.

## Data Availability

No datasets were generated or analysed during the current study.

## References

[1] Berman RA, Wurtz RH (2008) Exploring the pulvinar path to visual cortex. Prog Brain Res 171:467–473 18718342 10.1016/S0079-6123(08)00668-7 PMC2802535

[2] Berman RA, Wurtz RH (2010) Functional identification of a pulvinar path from superior colliculus to cortical area MT. J Neurosci 30(18):6342–6354 20445060 10.1523/JNEUROSCI.6176-09.2010 PMC2919315

[3] Burle B, Spieser L, Roger C, Casini L, Hasbroucq T, Vidal F (2015) Spatial and temporal resolutions of EEG: is it really black and white? A scalp current density view. Int J Psychophysiol 97:210–220 25979156 10.1016/j.ijpsycho.2015.05.004 PMC4548479

[4] Cortes N, de Souza BOF, Casanova C (2020) Pulvinar Modulates Synchrony across Visual Cortical Areas, *Vision* 4, 22 10.3390/vision4020022 PMC7357165 32290073

[5] Crabtree JW (2018) Functional diversity of thalamic reticular subnetworks. Front Syst Neurosci 12:41 30405364 10.3389/fnsys.2018.00041 PMC6200870

[6] Crouzet SM, Joubert OR, Thorpe SJ, Fabre-Thorpe M (2012) Animal detection precedes access to scene category. PLoS ONE 7(12):e51471 23251545 10.1371/journal.pone.0051471 PMC3518465

[7] Dehaene S, Changeux JP, Naccache L (2011) The Global Neuronal Workspace Model of Conscious Access: From Neuronal Architectures to Clinical Applications, *Springer-Verlag Berlin Heidelberg* 55–84

[8] Del Cul A, Baillet S, Dehaene S (2007) Brain dynamics underlying the nonlinear threshold for access to consciousness. PLoS Biol 5(10):e260 17896866 10.1371/journal.pbio.0050260 PMC1988856

[9] de Souza BOF, Cortes N, Casanova C (2020) Pulvinar modulates contrast responses in the Visual Cortex as a function of cortical hierarchy. Cereb Cortex 30(3):1068–1086 31408095 10.1093/cercor/bhz149 PMC7132966

[10] Di Lollo V (2012) The feature-binding problem is an ill-posed problem. Trends Cogn Sci 16(6):317–321 22595013 10.1016/j.tics.2012.04.007

[11] Doesburg SM, Roggeveen AB, Kitajo K, Ward LM (2008) Large-scale gamma-band phase synchronization and selective attention. Cereb Cortex 18(2):386–396 17556771 10.1093/cercor/bhm073

[12] Doesburg SM, Green JJ, McDonald JJ, Ward LM (2009) Rhythms of consciousness: binocular rivalry reveals large-scale oscillatory network dynamics mediating visual perception. PLoS ONE 4(7):e6142 19582165 10.1371/journal.pone.0006142 PMC2702101

[13] Enns JT, Di Lollo V (2000) What’s new in visual masking? Trends Cogn Sci 4(9):345–352 10962616 10.1016/S1364-6613(00)01520-5

[14] Fakche C, Dugué L (2024) Perceptual cycles travel across Retinotopic Space. J Cogn Neurosci 36(1):200–216 37902594 10.1162/jocn_a_02075

[15] Fiebelkorn IC, Pinsk MA, Kastner S (2018) A dynamic interplay within the Frontoparietal Network underlies rhythmic spatial attention. Neuron 99(4):842–853 30138590 10.1016/j.neuron.2018.07.038 PMC6474777

[16] Fries P, Nikolic D, Singer W (2007) The gamma cycle. Trends Neurosci 30(7):309–316 17555828 10.1016/j.tins.2007.05.005

[17] Fries P (2015) Rhythms for cognition. Communication through Coherence Neuron 88(1):220–235 26447583 10.1016/j.neuron.2015.09.034 PMC4605134

[18] Gautrais J, Thorpe S (1998) Rate coding versus temporal order coding: a theoretical approach. BioSystems 48(1–3):57–65 9886632 10.1016/S0303-2647(98)00050-1

[19] Gray CM, Di Viana G (1997) Stimulus-dependent neuronal oscillations and local synchronization in striate cortex of the alert cat. J Neurosci 17(9):3239–3253 9096157 10.1523/JNEUROSCI.17-09-03239.1997 PMC6573658

[20] Gregoriou GG, Gotts SJ, Zhou H, Desimone R (2009) High-frequency, long-range coupling between prefrontal and visual cortex during attention. Science 324(5931):1207–1210 19478185 10.1126/science.1171402 PMC2849291

[21] Han HB, Hwang E, Lee S, Kim MS, Choi JH (2017) Gamma-Band activities in Mouse Frontal and Visual Cortex Induced by coherent dot motion. Sci Rep 7:43780 28252109 10.1038/srep43780 PMC5333145

[22] Harris JA, Mihalas S, Hirokawa KE, Whitesell JD, Choi H, Bernard A, Bohn P, Caldejon S, Casal L, Cho A, Feiner A, Feng D, Gaudreault N, Gerfen CR, Graddis N, Groblewski PA, Henry AM, Ho A, Howard R, Knox JE, Kuan L, Kuang X, Lecoq J, Lesnar P, Li Y, Luviano J, McConoughey S, Mortrud MT, Naeemi M, Ng L, Oh SW, Ouellette B, Shen E, Sorensen SA, Wakeman W, Wang Q, Wang Y, Williford A, Phillips JW, Jones AR, Koch C, Zeng H (2019) Hierarchical organization of cortical and thalamic connectivity. Nature 575(7781):195–202 31666704 10.1038/s41586-019-1716-z PMC8433044

[23] Hopf JM, Schoenfeld MA, Heinze HJ (2005) The temporal flexibility of attentional selection in the visual cortex. Curr Opin Neurobiol 15(2):183–187 15831400 10.1016/j.conb.2005.03.008

[24] Jensen O, Bonnefond M, VanRullen R (2012) An oscillatory mechanism for prioritizing salient unattended stimuli. Trends Cogn Sci 16(4):200–206 22436764 10.1016/j.tics.2012.03.002

[25] Jensen O, Gips B, Bergmann TO, Bonnefond M (2014) Temporal coding organized by coupled alpha and gamma oscillations prioritize visual processing. Trends Neurosci 37(7):357–369 24836381 10.1016/j.tins.2014.04.001

[26] Jones EG (2002) Thalamic circuitry and thalamocortical synchrony. Philos Trans R Soc Lond B Biol Sci 357(1428):1659–1673 12626002 10.1098/rstb.2002.1168 PMC1693077

[27] Kaas JH, Baldwin MKL (2019) The evolution of the Pulvinar Complex in Primates and its role in the dorsal and ventral streams of cortical Processing. Vision 4(1):3 31905909 10.3390/vision4010003 PMC7157193

[28] Kahneman D, Treisman A, Gibbs BJ (1992) The reviewing of object files: object-specific integration of information. Cogn Psychol 24(2):175–219 1582172 10.1016/0010-0285(92)90007-O

[29] Kienitz R, Schmid MC, Dugué L (2022) Rhythmic sampling revisited: experimental paradigms and neural mechanisms. Eur J Neurosci 55(11–12):3010–3024 34643973 10.1111/ejn.15489

[30] Lestienne R, Gary-Bobo E, Przybyslawski J, Saillour P, Imbert M (1990) Temporal correlations in modulated evoked responses in the visual cortical cells of the cat. Biol Cybern 62(5):425–440 2331491 10.1007/BF00197649

[31] Lestienne R (2001) Spike timing, synchronization and information processing on the sensory side of the central nervous system. Prog Neurobiol 65(6):545–591 11728644 10.1016/S0301-0082(01)00019-3

[32] Liu Y, Paradis AL, Yahia-Cherif L, Tallon-Baudry C (2012) Activity in the lateral occipital cortex between 200 and 300 ms distinguishes between physically identical seen and unseen stimuli. Front Hum Neurosci 6:211 22848195 10.3389/fnhum.2012.00211 PMC3404546

[33] Markram H, Muller E, Ramaswamy S, Reimann MW, Abdellah M, Sanchez CA, Ailamaki A, Alonso-Nanclares L, Antille N, Arsever S, Kahou GA, Berger TK, Bilgili A, Buncic N, Chalimourda A, Chindemi G, Courcol JD, Delalondre F, Delattre V, Druckmann S, Dumusc R, Dynes J, Eilemann S, Gal E, Gevaert ME, Ghobril JP, Gidon A, Graham JW, Gupta A, Haenel V, Hay E, Heinis T, Hernando JB, Hines M, Kanari L, Keller D, Kenyon J, Khazen G, Kim Y, King JG, Kisvarday Z, Kumbhar P, Lasserre S, Le Bé JV, Magalhães BR, Merchán-Pérez A, Meystre J, Morrice BR, Muller J, Muñoz-Céspedes A, Muralidhar S, Muthurasa K, Nachbaur D, Newton TH, Nolte M, Ovcharenko A, Palacios J, Pastor L, Perin R, Ranjan R, Riachi I, Rodríguez JR, Riquelme JL, Rössert C, Sfyrakis K, Shi Y, Shillcock JC, Silberberg G, Silva R, Tauheed F, Telefont M, Toledo-Rodriguez M, Tränkler T, Van Geit W, Díaz JV, Walker R, Wang Y, Zaninetta SM, DeFelipe J, Hill SL, Segev I, Schürmann F (2015) Reconstruction and Simulation of Neocortical Microcircuitry. Cell 163(2):456–492 26451489 10.1016/j.cell.2015.09.029

[34] Maunsell JHR (2015) Neuronal mechanisms of vision. Ann Rev Vis Sci 24(1):373–391 10.1146/annurev-vision-082114-035431 PMC8279254 28532368

[35] Mountcastle VB (1997) The columnar organization of the neocortex. Brain 120:701–722 9153131 10.1093/brain/120.4.701

[36] Quax S, Jensen O, Tiesinga P (2017) Top-down control of cortical gamma-band communication via pulvinar induced phase shifts in the alpha rhythm. PLoS Comput Biol 13(5):e1005519 28472057 10.1371/journal.pcbi.1005519 PMC5436894

[37] Ray S, Maunsell JH (2010) Differences in gamma frequencies across visual cortex restrict their possible use in computation. Neuron 67(5):885–896 20826318 10.1016/j.neuron.2010.08.004 PMC3001273

[38] Ray S, Maunsell JH (2011) Different origins of gamma rhythm and high-gamma activity in macaque visual cortex. PLoS Biol 9(4):e1000610 21532743 10.1371/journal.pbio.1000610 PMC3075230

[39] Richmond BJ (2009) Stochasticity, spikes and decoding: sufficiency and utility of order statistics. Biol Cybern 100(6):447–457 19517130 10.1007/s00422-009-0321-x PMC2745726

[40] Roberts MJ, Lowet E, Brunet NM, Ter Wal M, Tiesinga P, Fries P, De Weerd P (2013) Robust gamma coherence between macaque V1 and V2 by dynamic frequency matching. Neuron 78(3):523–536 23664617 10.1016/j.neuron.2013.03.003

[41] Saalmann YB, Kastner S (2011) Cognitive and perceptual functions of the visual thalamus. Neuron 71(2):209–223 21791281 10.1016/j.neuron.2011.06.027 PMC3148184

[42] Saalmann YB, Pinsk MA, Wang L, Li X, Kastner S (2012) The pulvinar regulates information transmission between cortical areas based on attention demands. Science 337(6095):753–756 22879517 10.1126/science.1223082 PMC3714098

[43] Saalmann YB, Ly R, Pinsk MA, Kastner S (2018) Pulvinar influences parietal delay activity and information transmission between dorsal and ventral visual cortex in macaques, *bioRxiv* 405381

[44] Salami M, Itami C, Tsumoto T, Kimura F (2003) Change of conduction velocity by regional myelination yields constant latency irrespective of distance between thalamus and cortex. Proc Natl Acad Sci USA 100(10):6174–6179 12719546 10.1073/pnas.0937380100 PMC156345

[45] Schmid MC, Singer W, Fries P (2012) Thalamic coordination of cortical communication. Neuron 75(4):551–552 22920248 10.1016/j.neuron.2012.08.009

[46] Schmolesky MT, Wang Y, Hanes DP, Thompson KG, Leutgeb S, Schall JD, Leventhal AG (1998) Signal timing across the macaque visual system. J Neurophysiol 79(6):3272–3278 9636126 10.1152/jn.1998.79.6.3272

[47] Scholl LR, Foik AT, Lyon DC (2020) Projections between visual cortex and pulvinar nucleus in the rat. J Comp Neurol 529(1):129–140 32361987 10.1002/cne.24937 PMC7606250

[48] Sergent C, Baillet S, Dehaene S (2005) Timing of the brain events underlying access to consciousness during the attentional blink. Nat Neurosci 8:1391–1400 16158062 10.1038/nn1549

[49] Singer W, Gray CM (1995) Visual feature integration and the temporal correlation hypothesis. Annu Rev Neurosci 18:555–586 7605074 10.1146/annurev.ne.18.030195.003011

[50] Singer W (2007) Binding by synchrony. Scholarpedia 2(12):1657 10.4249/scholarpedia.1657

[51] Singer W (2013) Cortical dynamics revisited. Trends Cogn Sci 17(12):616–626 24139950 10.1016/j.tics.2013.09.006

[52] Sokoliuk R, VanRullen R (2016) Global and local oscillatory entrainment of visual behavior across retinotopic space. Sci Rep 6:25132 27126642 10.1038/srep25132 PMC4850391

[53] Spaak E, Bonnefond M, Maier A, Leopold DA, Jensen O (2012) Layer-specific entrainment of gamma-band neural activity by the alpha rhythm in monkey visual cortex. Curr Biol 22(24):2313–2318 23159599 10.1016/j.cub.2012.10.020 PMC3528834

[54] Spalek TM, Unnikrishnan KP, Di Lollo V (2023) Need for cross-level iterative re-entry in models of visual processing, *Psychon Bull* Rev. 2023 Oct 17 10.3758/s13423-023-02396-x PMC11192676 37848658

[55] Steriade M, Contreras D, Amzica F, Timofeev I (1996) Synchronization of fast (30–40 hz) spontaneous oscillations in intrathalamic and thalamocortical networks. J Neurosci 16(8):2788–2808 8786454 10.1523/JNEUROSCI.16-08-02788.1996 PMC6578775

[56] Tallon-Baudry C, Bertrand O, Delpuech C, Pernier J (1996) Stimulus specificity of phase-locked and non-phase-locked 40 hz visual responses in human. J Neurosci 16(13):4240–4249 8753885 10.1523/JNEUROSCI.16-13-04240.1996 PMC6579008

[57] Tallon-Baudry C, Bertrand O (1999) Oscillatory gamma activity in humans and its role in object representation. Trends Cogn Sci 3(4):151–162 10322469 10.1016/S1364-6613(99)01299-1

[58] Thomson AM, Bannister AP (2003) Interlaminar connections in the neocortex. Cereb Cortex 13(1):5–14 12466210 10.1093/cercor/13.1.5

[59] Thomson AM, Lamy C (2007) Functional maps of neocortical local circuitry. Front Neurosci 1(1):19–42 18982117 10.3389/neuro.01.1.1.002.2007 PMC2518047

[60] Thomson AM (2010) Neocortical layer 6, a review. Front Neuroanat 4:13 20556241 10.3389/fnana.2010.00013 PMC2885865

[61] Tiesinga PH, Fellous JM, Salinas E, José JV, Sejnowski TJ (2004) Inhibitory synchrony as a mechanism for attentional gain modulation. J Physiol 98(4–6):296–314 10.1016/j.jphysparis.2005.09.002 PMC2872773 16274973

[62] Treisman AM, Gelade G (1980) A feature-integration theory of attention. Cogn Psychol 12(1):97–136 7351125 10.1016/0010-0285(80)90005-5

[63] Van Essen DC, Anderson CH, Felleman DJ (1992) Information processing in the primate visual system: an integrated systems perspective. Science 255(5043):419–423 1734518 10.1126/science.1734518

[64] Van Essen DC, Anderson CH (1995) Information processing strategies and pathways in the primate visual system. In: Zornetzer SF, Davis JL, Lau C, McKenna T (eds) An introduction to neural and electronic networks, 2nd edn. Academic, pp 45–76

[65] van Kerkoerle T, Self MW, Dagnino B, Gariel-Mathis MA, Poort J, van der Togt C, Roelfsema PR (2014) Alpha and gamma oscillations characterize feedback and feedforward processing in monkey visual cortex. Proc Natl Acad Sci U S A 111(40):14332–14341 25205811 10.1073/pnas.1402773111 PMC4210002

[66] VanRullen R, Guyonneau R, Thorpe SJ (2005) Spike times make sense. Trends Neurosci 28:1–4 15626490 10.1016/j.tins.2004.10.010

[67] VanRullen R, Dubois J (2011) The psychophysics of brain rhythms. Front Psychol 2:203 21904532 10.3389/fpsyg.2011.00203 PMC3163286

[68] Veit MJ, Kucyi A, Hu W, Zhang C, Zhao B, Guo Z, Yang B, Sava-Segal C, Perry C, Zhang J, Zhang K, Parvizi J (2021) Temporal order of signal propagation within and across intrinsic brain networks. Proc Natl Acad Sci USA 118(48):e2105031118 34819365 10.1073/pnas.2105031118 PMC8640784

[69] von der Malsburg C, Schneider W (1986) A neural cocktail-party processor. Biol Cybern 54(1):29–40 3719028 10.1007/BF00337113

[70] Wehr M, Zador AM (2003) Balanced inhibition underlies tuning and sharpens spike timing in auditory cortex. Nature 426(6965):442–446 14647382 10.1038/nature02116

[71] Wilent WB, Contreras D (2004) Synaptic responses to whisker deflections in rat barrel cortex as a function of cortical layer and stimulus intensity. J Neurosci 24(16):3985–3998 15102914 10.1523/JNEUROSCI.5782-03.2004 PMC6729426

[72] Youssofzadeh V, Prasad G, Fagan AJ, Reilly RB, Martens S, Meaney JF, Wong-Lin K (2015) Signal propagation in the human visual pathways: an effective connectivity analysis. J Neurosci 35(39):13501–13510 26424894 10.1523/JNEUROSCI.2269-15.2015 PMC6605472

